# Gross morphology and adhesion-associated physical properties of *Drosophila* larval salivary gland glue secretion

**DOI:** 10.1038/s41598-024-57292-8

**Published:** 2024-04-29

**Authors:** Milan Beňo, Denisa Beňová-Liszeková, Ivan Kostič, Michal Šerý, Lucia Mentelová, Michal Procházka, Ján Šoltýs, Ludmila Trusinová, Mário Ritomský, Lubomír Orovčík, Monika Jerigová, Dušan Velič, Peter Machata, Mária Omastová, Bruce A. Chase, Robert Farkaš

**Affiliations:** 1 Laboratory of Developmental Genetics, Institute of Experimental Endocrinology, Biomedical Research Center v.v.i., Slovak Academy of Sciences, Dúbravská Cesta 9, 84505 Bratislava, Slovakia; 2 Department of Sensor Information Systems and Technologies, Institute of Informatics v.v.i., Slovak Academy of Sciences, Dúbravská Cesta 9, 845 07 Bratislava, Slovakia; 3 Department of Applied Physics and Technology, Faculty of Education, University of South Bohemia, Jeronýmova 10, 37115 České Budějovice, Czech Republic; 4 Department of Genetics, Comenius University, Mlynská Dolina, B-1, 84215 Bratislava, Slovakia; 5 Department of Composite Materials, Polymer Institute v.v.i., Slovak Academy of Sciences, Dúbravská Cesta 9, 84541 Bratislava, Slovakia; 6 Department of Physics and Technology at Nanoscale, Institute of Electrical Engineering v.v.i., Slovak Academy of Sciences, Dúbravská Cesta 9, 84104 Bratislava, Slovakia; 7 Division of Microstructure of Surfaces and Interfaces, Institute of Materials and Machine Mechanics v.v.i., Slovak Academy of Sciences, Dúbravská Cesta 9, 84513 Bratislava, Slovakia; 8 Laboratory of Secondary Ion Mass-Spectrometry, International Laser Centre, Slovak Centre of Scientific and Technical Information, Ilkovičova 3, 84104 Bratislava, Slovakia; 9 Department of Biology, University of Nebraska, 6001 Dodge Street, Omaha, NE 68182-0040 USA; 10 Department of Data Analytics, Endeavor Health, NorthShore University Health System, Skokie, IL 60077 USA

**Keywords:** Pupariation, *Drosophila*, Larval salivary glands, Exocytotic salivary glue secretion, Glue-substrate relationship, Triboelectric series, Cell signalling, Morphogenesis, Atomic force microscopy, Electron microscopy, Biomechanics

## Abstract

One of the major functions of the larval salivary glands (SGs) of many *Drosophila* species is to produce a massive secretion during puparium formation. This so-called proteinaceous glue is exocytosed into the centrally located lumen, and subsequently expectorated, serving as an adhesive to attach the puparial case to a solid substrate during metamorphosis. Although this was first described almost 70 years ago, a detailed description of the morphology and mechanical properties of the glue is largely missing. Its main known physical property is that it is released as a watery liquid that quickly hardens into a solid cement. Here, we provide a detailed morphological and topological analysis of the solidified glue. We demonstrated that it forms a distinctive enamel-like plaque that is composed of a central fingerprint surrounded by a cascade of laterally layered terraces. The solidifying glue rapidly produces crystals of KCl on these alluvial-like terraces. Since the properties of the glue affect the adhesion of the puparium to its substrate, and so can influence the success of metamorphosis, we evaluated over 80 different materials for their ability to adhere to the glue to determine which properties favor strong adhesion. We found that the alkaline Sgs-glue adheres strongly to wettable and positively charged surfaces but not to neutral or negatively charged and hydrophobic surfaces. Puparia formed on unfavored materials can be removed easily without leaving fingerprints or cascading terraces. For successful adhesion of the Sgs-glue, the material surface must display a specific type of triboelectric charge. Interestingly, the expectorated glue can move upwards against gravity on the surface of freshly formed puparia via specific, unique and novel anatomical structures present in the puparial’s lateral abdominal segments that we have named bidentia.

## Introduction

Bioadhesion is a widespread biological adaptation that living organisms use to adhere to different surfaces by using natural macromolecules. Fundamentally, bioadhesion is the phenomenon whereby biologically produced cementing materials adhere to different surfaces. It has been described in microorganisms, plants, and both invertebrate and vertebrate animals^1–3^. Since bioadhesion is critical to many life-history strategies, studying it in a well-characterized developmental- and molecular-genetic model system can provide considerable insight into how its use has been fine-tuned during evolution. Such studies are likely to uncover unanticipated relationships between the biological production of the cement and life-cycle success. This rationale prompted us to investigate the bioadhesive properties of the glue produced by the larval salivary glands (SGs) of *Drosophila melanogaster*. These glands are exemplary tubular and single-layered paired epithelial exocrine organs of ectodermal origin^4–6^. A small group of about eight proteins that are uniquely and strongly expressed in the SGs of late last instar larvae, are secreted into a centrally located gland lumen to form a glue^7–11^ which affixes the freshly forming puparium to a substrate during the initial phases of metmorphosis^6,12,13^. The SG glue is produced in the form of secretory granules which constitute the components of the **s**alivary **g**lue **s**ecretion (Sgs). The Sgs proteins form a highly specialized and unique extracellular composite glue matrix.

At the end of the last larval instar, the slowly elevating titer of the steroid hormone ecdysone, which is released into circulation from the ring gland, induces a complex response that leads to the initiation of metamorphosis^14–17^. In the SG tissue, this is mirrored by the development of large euchromatic interecdysial puffs in the larva’s giant polytene chromosomes at the complex genetic loci that encode some of the glue proteins. Their appearance reflects a cascade of transcriptional regulation that is followed by the secretion of the glue by exocytosis. The biochemical, molecular and genetic analysis of the *Sgs* genes and their proteins led to a detailed understanding of their molecular genetic regulation and the exocytotic release of the glue in response to ecdysone, providing a seminal model for understanding developmentally regulated gene expression^18–23^.

Previous research focused intensely on the developmental control of glue release. That likely led to assumption that the only major and unambiguously documented function of the larval SGs is production and release of Sgs-glue, as was believed for several decades. In the past several years, however, work from our group has demonstrated that the SGs also provide another fundamentally important function: following the release of the glue but prior to pupation, the prepupal SGs utilize apocrine secretion to deliver multiple components to the exuvial fluid that lies between the metamorphosing pupae and its chitinous case^6,24–27^. The relationship between glue production and production of exuvial fluid is only partially understood.

Transmission electron microscopy (TEM) revealed that prior to Sgs-glue release, Sgs-secretory granules have three different components: (1) a fine particulate dense component, (2) a filamentous-like paracrystalline component, and (3) a foamy electron-transparent component^28–30^. Following exocytosis, the material released from the granules appears to be an amorphous homogenous electron-dense matrix with scattered lighter (electron-lucent) rosettes^28–32^.

Recently, we used scanning electron microscopy (SEM) to characterize the surface and internal substructure of the exocytosed, and then the expectorated and solidified secretion^33^. This is characterized by an internal spongious-to-trabecular infrastructure that reflects the dynamically changing state of glue hydration. The expectorated and solidified glue possesses a micellar fibrous-like spongious infrastructure with „fibers “ of highly irregular thicknesses. Furthermore, we found that in addition to its bioadhesive properties, the Sgs is highly efficient at gluing and trapping microorganisms, suggesting that it may contribute to a defense role. This led to the intriguing hypothesis that the mechanical and bioadhesive properties of the glue are tuned to function in concert with the exuvial fluid produced by apocrine secretion to enhance the survival of *Drosophila* pupae in particular environments.

Until now, the Sgs has been considered to be a water-soluble glue, perhaps because established protocols use a drop (10–50 µl) of water to facilitate the removal and collection of prepupae and pupae from the glass or plastic walls of culture (fly) vials. This view was based on the misconception that the detachment was due to the quick solubilization of the Sgs-glue that affixes the pupariating larva to a substrate^6^. We show here that upon removal of the puparial case from the fly vial, the solidified Sgs-glue remains more-or-less stable and unchanged after a brief treatment with water. We initially speculated that perhaps only very little (less than 1%) of the Sgs-glue becomes solubilized by water, and that this was sufficient to easily remove the puparium or pupa from the wall of the vial. During the present study, however, we discovered that the major factor contributing to the relatively easy removal of the puparial case from the vial wall by a drop of water is the dissolution of agar-yeast-cornmeal food contaminants which the wandering larva retains on its surface.

With the molecular and genetic tools available in *Drosophila*, *e*.*g*. mutations, RNAi knockdown, DNA editing, it is feasible to study the importance of individual Sgs-proteins to the function of the Sgs-glue. There have been, however, no investigations to establish reference parameters for such studies. In contrast to the very limited information available on the physical properties of the *Drosophila* Sgs-glue, numerous and detailed studies have described the physical properties of the silk produced by the silk glands of butterfly caterpillars (silkmoths) and spiders, which are strongly orthologous organs to the *Drosophila* SGs. Since these studies have been instrumental in understanding how silk properties function within these organisms life-history strategies, the characterization of the basic mechanical properties of the Sgs-glue in *Drosophila* is a prerequisite to future studies where genetic tools could be used to develop deeper insights into these questions. Therefore, in the present paper we provide reference parameters for such comparative studies. In this report, we describe (1) the morphology and topography of the Sgs-glue after its expectoration and solidification by using light microscopy, SEM, and atomic force microscopy (AFM); (2) the mechanical properties of the glue, including its adhesiveness to different materials (tensiometry), that uncovered which surfaces are preferred for strong adhesion; (3) the stiffness, hardness, and elasticity of the Sgs-glue, evaluated using nanoindentation and compared to other types of materials; and (4) the previously unknown anatomical structure in larvae and puparia that is directly associated with gluing the puparial case to its substrate. These results will allow for the investigation of the specific role of individual Sgs-proteins/genes in *D*. *melanogaster* to understand their evolution across more or less distant *Drosophila* species.

## Results

### Description of expectorated and solidified Sgs-glue

**Figure 1:**
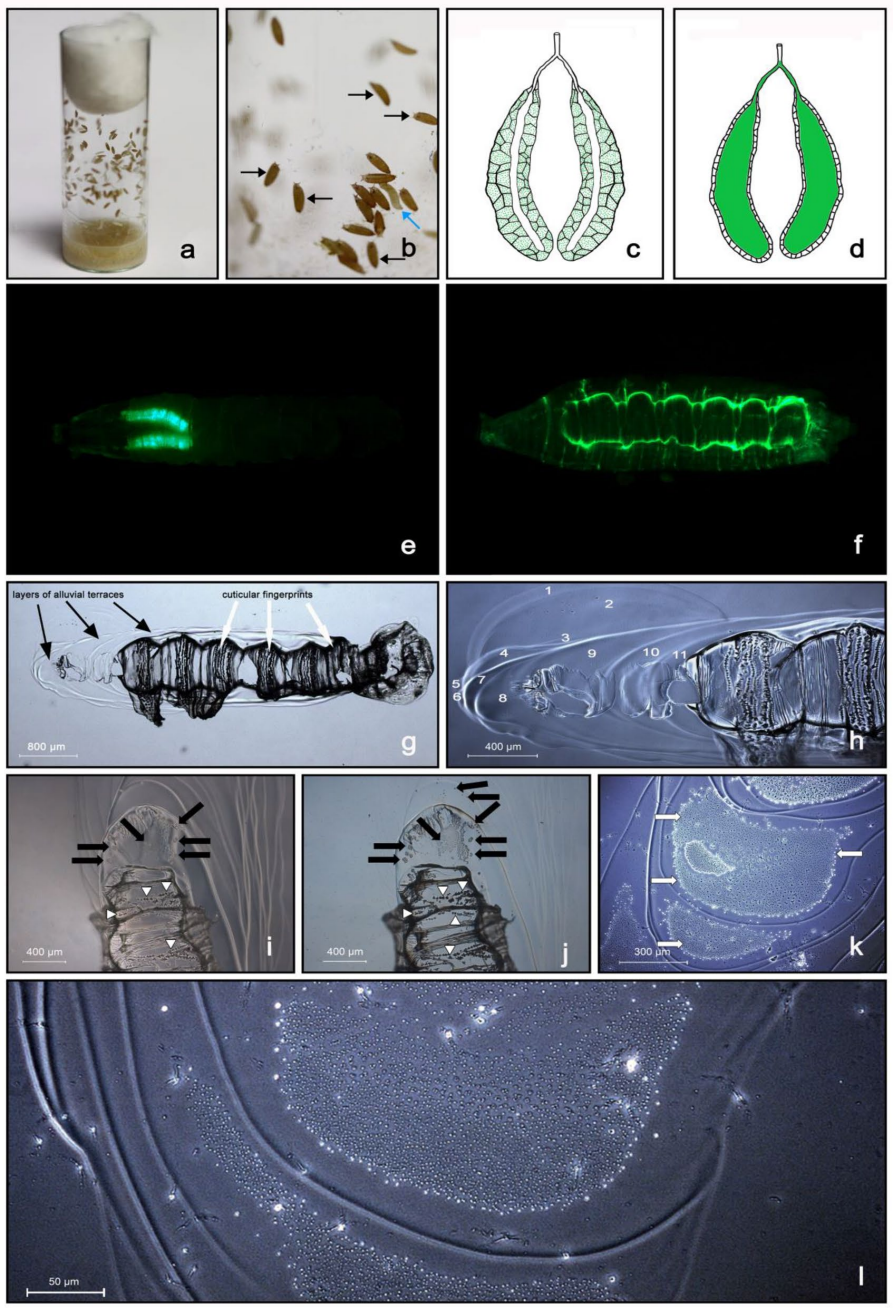
The Sgs-glue that is produced in the larval SGs becomes released during pupariation beneath the body cuticle and its vicinity to form a solidified plaque. (**a**) Glass culture vial with mature last-instar larvae leaving the food and climbing up in search of optimal pupariation sites. (**b**) Detailed view of a culture vial with wandering white larvae (blue arrows) and immobile puparia (black arrows) that are cemented to the glass wall by using excreted Sgs-glue. (**c**) Late third instar larval SGs from the end of the feeding period, the cytoplasm of which is full of secretory granules (green dots). (**d**) Third instar larva in wandering period released by exocytosis the secretory granules observed in (**c**) into the gland lumen, which becomes extremely wide (green signal corresponding to Sgs3-GFP protein). In this way, the Sgs-glue secretion becomes ready for abrupt expectoration during pupariation. (**e**) Late third instar larva before initiating the wandering period, in which the SGs (green fluorescent signal) were labeled by transgenic *Sgs3*-*GFP* construct, the expression of which is tissue-specific for this organ. (**f**) Freshly formed puparium shortly after expectoration of the glue labeled with Sgs3-GFP protein. The green signal highlights the quickly solidified glue plaque which comprises two elementary structures: a strongly fluorescent fingerprint, and less fluorescent layers of cascading terraces. Medium-strong GFP signal identifies the borders of individual abdominal segments. (**g**) Light microscopy view (wide field) of the released and solidified Sgs-glue, which helps to distinguish between the alluvial-like cascades (or terraces) of the Sgs-glue, and the cuticular fingerprints imprinted in the glue. (**h**) Phase contrast view of the detail of the anterior portion of the Sgs-glue plaque showing numerous (1 to 11) layers of cascading terraces. (**i**) Under Nomarski interference optics the layered terraces closest to the fingerprint indicated the presence of previously unnoticed structures (black arrows) that are optically distinct from the smooth surface of the plaque. White arrowheads point to well-preserved „fingerprints “ of the cuticular denticle belts on the ventral side of the puparial case. (**j**) These elements have a granular texture and a different composition than their surroundings that became even more apparent under bright field illumination with a lowered condenser (black arrows). Some of them are isolated individual structures, some may constitute dense areas. White arrowheads point to the fingerprints of denticle belts. (**k**) Slightly magnified view of a selected area of layered cascading terraces under phase contrast revealed that they contain dense territories with a grainy surface (white arrows) with apparently different shifts of the scattered light at the area ‘s border. (**l**) Detailed view of a similar area under phase contrast with a lowered condenser indicating that the grainy surface has a distinguished topography that reaches above the surrounding smooth layer of the glue.

After completing larval development throughout three instars, the mature larva leaves its source of food and seeks an appropriate place for pupariation. In the laboratory, the white-colored larvae enter the wandering stage and many can be observed on the glass wall of culture vials (Fig. 1a). At the moment of pupariation, when all motion stops, endocrine conditions initiate sclerotization and tanning of the puparial cuticle, and within 1 to 1.5 h, the puparia turn brownish (Fig. 1b). The Sgs secretion is released just prior to pupariation, when the larva becomes motionless, except in the head: during the last 30–120 s prior to complete immobilization, the larva expectorates the secretion accumulated inside its SG lumen (Fig. 1c–e). At this stage, the larval abdomen suddenly performs several fast repeated waves of posterior-to-anterior peristaltic movements, and subsequently the larval head makes several semicircular movements around the thorax while the mouth pulsates back and forth, generating a sort of pumping force to help to expel a clear jelly-like fluid usually in 4 to 10 consecutive doses. The fluid is poured in the immediate vicinity of the larva and beneath the body cuticle in the form of a viscoelastic gel (Fig. 1f). The emitted colorless fluid suddenly hardens (in 3 to 10 s, depending on ambient humidity) and, after the final dose is released, it glues the freshly formed puparium to the substrate. In contrast to previous expectations, the behavior of the larval head, specifically its semicircular movements during the stepwise consecutive release of the secretory fluid in the last moments before complete immobilization, results in the formation of an enamel-like plaque with alluvial-like cascades or terraces in the Sgs-glue formed in the closest vicinity of the puparium (Fig. 1g,h). These layered terraces of the glue plaque appear smooth under the stereomicroscope; however, closer inspection under a bright field light microscope or using phase contrast and Nomarski interference optics, revealed the presence of numerous (hundreds to thousands) of small previously unobserved particles inside their shallow valleys (Figs. 1i–l). The hardening of the glue was so fast, that the white puparium could only be safely removed with forceps only within the first 10 to 20 s, otherwise the animal would be seriously injured. Upon this fast (immediate or early) removal of the puparium, we could observe in the places where it was cemented to a substrate with Sgs-glue, that there were perfect fingerprints of the ventral larval cuticle mirroring the individual segments (tergites) including the denticle belts (Fig. 1g–j). These fingerprint areas never contained the numerous small particles seen inside the alluvial-like layered terraces of the glue plaque.

**Figure 2:**
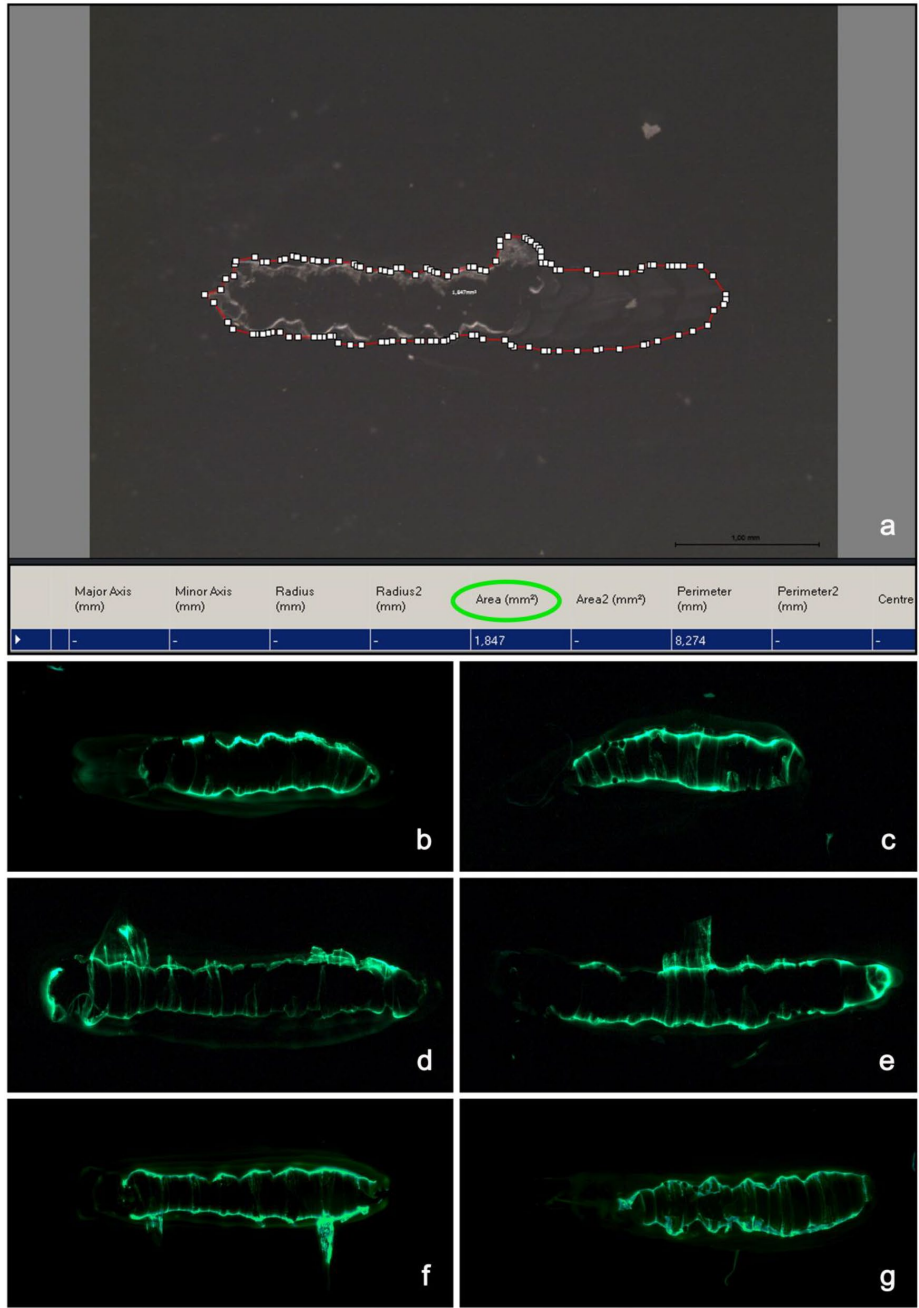
Imaging and measurement of glue plaque areas from individual animals. (**a**) Larvae were allowed to pupariate on a clean microscope glass slide where they expectorated their SG glue. As described under Materials and Methods, the freshly formed immobile puparium was removed within 10 to 20 s. Spilled plaque of wild-type and GFP-labeled glue were then photographed under a Leica DMR-E or Leica DM6000B microscope or Leica MZ16F-X/A stereomicroscope, and the glue area was measured using the Interactive Measurements module of the Leica LAS software. Several hundreds of spilled and solidified Sgs-glue plaques were measured. As illustrated in fluorescence images (**b**) to (**g**) from *Sgs3*-*GFP* transgenic larvae, the area occupied by the Sgs-glue plaque varies from animal to animal. In addition to the area occupied by the plaque, also the number of cascading terraces and the area of the cuticular fingerprint were variable. By subtracting these genuine fingerprint areas from the values of total plaque areas, we revealed that the area which the puparia utilize for contact with the released glue varies between 10 and 68%, with an average value of 42%. Some fingerprints displayed more prominent ventral segments with more visible denticle belts than others. Samples in (**d**), (**e**), and (**f**) show side-oriented pieces of skinny Sgs3-GFP that were formed during the removal of puparia from solidifying glue. Observations provided evidence that there is no universal or standard area that is occupied by the solidified glue, and therefore Sgs-glue mediates tangency (contact) of the puparial case with a substrate via apparent but not nominal contact area, and that this area must be measured individually.

We collected hundreds of solidified Sgs-glue samples released onto the glass surface of the microscope slides, took microscope images and, using the Interactive Measurements modul of the Leica LAS software, measured the area on which the glue secretion was spilled (Fig. 2a). The entire area on which solidified glue could be seen ranged from 0.586 to 4.523 mm^2^, varying most frequently from 0.851 to 3.309 mm^2^, with a median value close to 2.5 mm^2^. Interestingly, those areas where fingerprints of ventral cuticular pattern occurred and did not reach the smooth alluvial-like layered terraces, ranged from 0.390 to 1.254 mm^2^, indicating that the prepupa utilizes only between 12 and 56% of the released glue for active contact. In a few cases, it reached 68%, but the most frequent value was around 42%. The distribution of area sizes for Sgs-glue plaque and for fingerprints is depicted in histograms in Supplemental Fig. 1. As it could be seen from the series of fluorescence images obtained from the *Sgs3*-*GFP* transgenic individuals, the area occupied by the Sgs-glue plaque varied from animal to animal (Fig. 2b–g). Although these values are interesting and reproducible, they should be considered in an additional context. This is that the thickness of the glue plaque may also play a role in fixing the animal to a particular surface. Based on these observations, there is no universal or standard area which is occupied by the solidified glue and, therefore, Sgs-glue mediates tangency (contact) of the puparial case with a substrate via an apparent but not nominal contact area that must be measured individually. This indicates that in nature even relatively small contact area appears to be sufficient for efficient adhesion under most circumstances.

A unique view of Sgs-glue mediated adhesion was obtained by employing the *Sgs3*-*GFP* transgenic construct, which tissue-specifically expresses jellyfish green fluorescent protein (GFP) fused to Sgs3 protein and so becomes part of the released and solidified glue. The *Sgs3*-*GFP* construct is a fusion of the jellyfish GFP coding sequence inserted behind the 1.8 kb of the *Sgs3* gene carrying its upstream regulatory regions and the first third of its protein coding sequence truncated after nine tandem repeats^34^. For more details see Materials and Methods (in Supplement). As seen from the fluorescence images of released and solidified Sgs-glue (see Fig. 2b–g), the majority of the GFP signal (and in turn the majority of the Sgs3 protein) was present within the cuticular fingerprint (seal), whereas the alluvial cascades (terraces) of the glue, which are apparently thicker than the fingerprints, contained much less of this protein. By morphological appearance and consistency, there seemed to be a difference in the structure of glue plaque *vs*. cuticular fingerprint. The unequal distribution of the Sgs3 protein between these two may indicate that the secreted Sgs-glue produces two inherently distinct forms of the cement: (1) near homogenous and smooth plaque, and (2) structurally articulated fingerprint. This may potentially reflect two different levels or types of glue solidification.

Another remarkable feature of this solidified glue is that the GFP signal remained strong for number of weeks or even months (the longest period we tested was 22 months), even after repeated and prolonged illumination under 488 nm light. Under native conditions, the GFP signal from fused proteins decays in minutes (no longer than in 1 h) upon dissecting SGs in culture medium or upon the death of the larva. Similarly, the GFP signal becomes significantly reduced even under standard histochemical tissue fixation with 4% paraformaldehyde, and usually requires enhanced detection by anti-GFP antibody amplified via fluorochrome-conjugated secondary antibodies. Thus, the extremely well-preserved GFP signal shining inside the solidified glue indicates its strong structural stabilization.

### AFM and SEM studies reveal crystals in the Sgs-glue (plaque)

**Figure 3:**
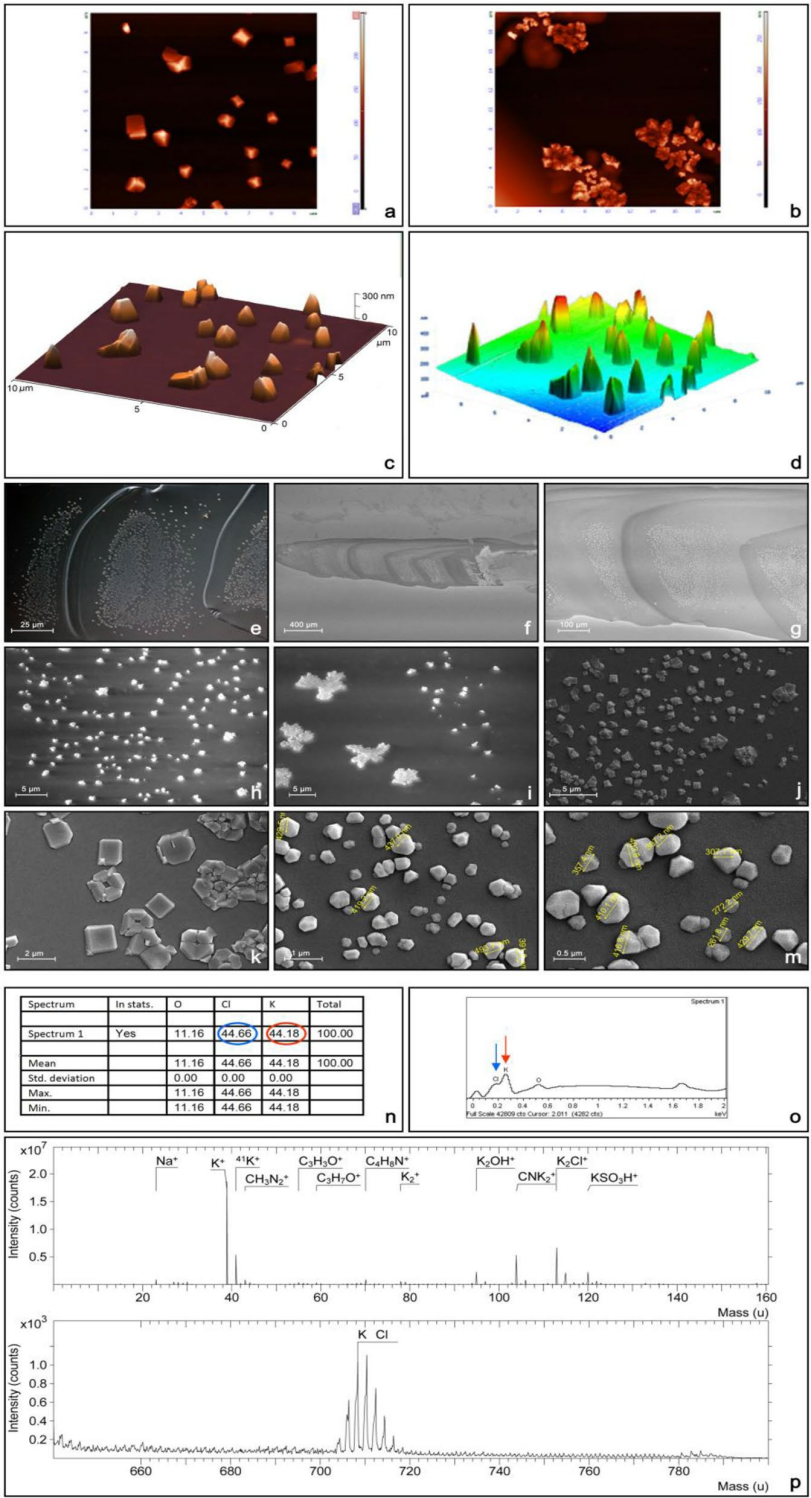
Topography of Sgs-glue surface and crystal composition. (**a**) and (**b**) AFM of the surface of alluvial-like terraces of solidified glue revealed that the grainy material is an ordered array of crystals in its nature (very light brown color). These crystal-like structures can be individual specimens or irregularly scattered groups in the form of clusters on the surface of otherwise smooth solidified glue (dark brown background). (**c**) and (**d**) Using 3D projection of Nova 1.1.0 or MDT Image Analysis 2.1.0 softwares, it could be seen that the otherwise smooth surface of the glue displayed some irregularities in the range of 5 to 300 nm (**c**), or even 450 nm as revealed by color depth coding in (**d**). However, individual crystals reached the size of 0.1 to 1.0 µm. (**e**) Detailed phase contrast view of the Sgs-glue plaque (similar to that in Fig. 1i and Fig. 1j). Each individual terrace had a different number of „grains “, some of which were aggregated into an archipelago-like arrangement, and some were randomly scattered. (**f**) and (**g**) SEM views (environmental mode) of anteriorly spread alluvial-like terrace cascades of Sgs-glue plaque at 150 × and 500 × magnification, respectively, showing dense areas with numerous crystals. These images demonstrated that the crystal structures were present on all terraces of the plaque with higher density in the anterior portion of the terrace (anterior is to the left in **e** to **g**). (**h**) to (**k**) More detailed SEM-views (magnifications from 8 000 to 30 000 ×) of crystals on the surface of solidified glue documenting that the majority of the crystals were individual grains although some were arranged in groups. (**l**) and (**m**) images document that the size of the majority of these crystals varied from 200 to 400 nm. However, individual crystals could range from 50 to 2000 nm. To measure the dimensions of the crystals, the measurement/annotation tool of FEI xTm software v. 6.2.8 was used. Both AFM and SEM clearly showed that crystals belonged to the face-centered cubic structural category with octahedral-like coordination geometry. (**n**) Raw data of spectral analysis of the crystals on the surface of the Sgs-glue plaque, obtained from Jeol JSM 7600F SEM microscope equipped with energy dispersive spectroscopy (EDS) and electron backscattered diffraction (EBD) detectors. Major spectral signals refered to potassium (K^+^) and chloride (Cl^-^) ions (in red circles). (**o**) Mass spectrogram from EDS detector of Jeol JSM 7600F SEM microscope. The red arrow points to the K^+^ signal, while the blue arrow points to the Cl^-^ signal. (**p**) A second independent method to confirm the identity of the crystals formed on the surface of the Sgs-glue plaque was secondary ion mass spectrometry (SIMS) using an Ion-TOF–SIMS IV spectrometer. The spectral intensity and mass units revealed the presence of potassium chloride (KCl) as the major component of these crystals. HCO_3_^-^, KOH, K_2_SO_4_ H^+^, K_3_SO_3_^+^, K_3_SO_4_^+^, CH_4_N^+^, and NH_4_ were identified as minor signals. Images (**f**) and (**g**) are SEM views under the environmental mode, whereas (**h**) to (**m**) are views of critically point dried and sputter-covered samples.

The finding of numerous small particles (grains) in Sgs-glue prompted us to examine the solidified glue under an atomic force microscope (AFM) and scanning electron microscope (SEM) making focused observations on the alluvial-like layered terraces. As illustrated in Fig. 3, simple AFM scanning of the Sgs-glue surface in contact mode [using a standard 65–130 kHz (HG:NSC36/AIBS) tip] revealed the presence of regular individual crystal-like structures (Fig. 3a,b) or clusters of crystals (Fig. 3c,d). Based on 3D imaging topography, the lateral size of crystals varied from 0.1 to 1 µm (Fig. 3d,e). To highlight a group of crystals and their closely-related sizes from a representative 10 × 10 µm scanned area, depth-code coloring was used (Fig. 3d). In a further step, we used this same sample mounted on a piece of microscope glass slide to view under SEM using an environmental mode. This approach identified the presence of numerous crystals, or groups of crystals, on areas of alluvial-like layered terraces with 50 to 1300 crystals in each of these areas (Fig. 3f–j). Further SEM measurements performed on critically point dried and sputter-covered samples, confirmed that the usual size of crystals was between 0.1 and 1 µm, but the great majority of them were around 0.3 to 0.6 µm. Rarely, a few crystals were below 0.05 or above 2.0 µm (Fig. 3k–m). Supplemental Fig. 2 shows a histogram with the distribution of crystal sizes. Although larger areas of terraces usually had more crystals than smaller ones, we did not find any clear correlation between these two variables. Both AFM and SEM clearly showed that the crystals belong to the face-centered cubic structural category with octahedral-like coordination geometry.

### Crystal determination on the surface of the Sgs-glue plaque

We then sought to identify the nature of these surprising and previously unannotated crystal aggregates. Considering the extraordinary speed of glue solidification (a few seconds after expectoration), we reasoned that the process of crystallization must be equally fast. Therefore, the identification of the crystal’s nature is pivotal for understanding their function. For this purpose, we used energy dispersive spectroscopy (EDS) and electron backscattered diffraction (EBD) detectors connected to a Jeol JSM 7600F SEM, and secondary ion mass spectrometry (SIMS) on an Ion-TOF–SIMS IV. Based on the spectral intensity and mass units, the majority of these crystals were identified as pure potassium chloride (KCl) (Fig. 3n–p). Indeed, the above SEM-identified face-centered cubic structure is typical for KCl crystals. The very short period for glue expectoration and extremely fast formation of crystal aggregates, indicates that the Sgs-solute must already possess a high concentration of KCl which most probably crystallizes due to solidification and/or rapid water-based solvent evaporation. With to its higher sensitivity, SIMS was able to identify minor components that included K_2_SO_4_, K_2_SO_3_, K_3_SO_4_, CH_4_N^+^, and KOH (Fig. 3p).

### Nanoindentation of Sgs-glue

**Figure 4:**
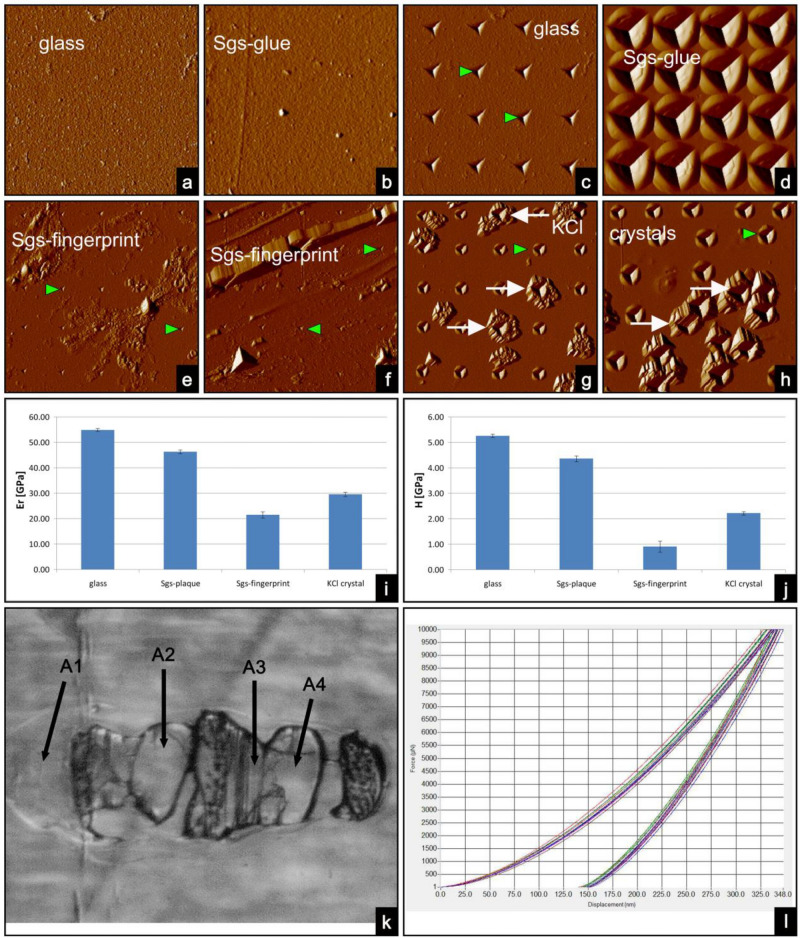
Determination of elastic modulus and hardness of Sgs-glue matrix by nanoindentation using a Hysitron TI 750D apparatus. (**a**) The overall appearance of the microscope glass surface was slightly less smooth than that of the glue-plaque (**b**), except for random crystals. For these measurements, the glue samples with fewer KCl crystals were purposely preselected under a light microscope. Using standard indentation parameters (25 °C and 35% relative humidity, scan rate 0.5 Hz, tip velocity 40 µm/s, setpoint 2 µN, and integral gain 240), the glass surface (**c**) allowed shallower indentations (green arrowheads) to be made than those visible in the smooth areas of the Sgs-glue plaque (**d**). Interestingly, nanoindentation against the fingerprint portion of the glue (**e**, **f**) produced very shallow indentations (green arrowheads), comparable to a clean glass surface, indicating that the fingerprint area contains much less glue mass than areas of cascading terraces. (**g**) and (**h**) On the contrary, nanoindentation against dense crystal areas revealed that KCl crystals (white arrows) are apparently softer than the surrounding Sgs-glue (green arrowheads). Elastic modulus (**i**) and relative hardness (**j**) of the glass, Sgs-plaque, Sgs-fingerprint and KCl crystals (expressed in GPa). Numerical values clearly document that the Sgs-plaque was almost as hard as glass, and also showed comparable elasticity. Although KCl crystals were softer than glass and the Sgs-plaque, and also displayed lower elasticity, their values were higher than that of Sgs-fingerprints. This paradox, when compared against nanoindentation images (**e**, **f**), can be explained by the presence of relatively free pieces of the Sgs-glue material that is not firmly in contact with the glass support and so provides a soft response to the nanoindenter tip. (**k**) Left: Light microscopy image of one of the selected samples showing different areas (A-1 to A-4) where nanoindentation measurements were performed. Right: an associated image of a nanoindentation. (**l**) Thirteen force *vs*. displacement nanoindentation curves measured on A-1. This picture represents a general example of nanoindentation curves measured from a single specific area (A-1) but curves had a very similar appearance also in other measured areas. These curves showed a good agreement, which means that the measurement area was homogeneous.

In order to gain more insights into the mechanical properties of the Sgs-glue we employed nanoindentation, a simple method that consists essentially of touching the material to determine mechanical properties that include elastic modulus and hardness. The maximum depth of penetration for a particular load, together with the slope of the unloading curve measured at the tangent to the data point at maximum load, leads to a measure of both hardness and elastic modulus of the specimen. For a complete comparison of the glue properties, we tested hardness (H) and relative elasticity (E_r_) in Sgs-plaque, fingerprint, and KCl crystals, and compared them with the glass on which the Sgs-glue was deposited. Using a three-sided Berkovich diamond tip (at 25 °C and 35% relative humidity, scan rate 0.5 Hz, tip velocity 40 µm/sec, setpoint 2 µN, and integral gain 240), a minimum of 10 indentations (usually 13 to 16) were performed on an area of 40 × 40 µm. The appearance of the surface of the Sgs-plaque by a Hysitron TI 750D indenter was slightly smoother than that of the microscope glass slide (Fig. 4a,b). The hardness of smooth Sgs-plaque varied between 4 and 4.7 GPa, whereas relative elasticity varied between 40 and 52 GPa. The surface of the microscope glass slide had a hardness of 5.0 to 5.5 GPa, and relative elasticity of 54 to 55 GPa (Fig. 4c,d,i,j), which appear unexpectedly close to Sgs-glue. As anticipated, the hardness of the Sgs-fingerprint area was much lower and varied between 0.5 and 1.3 GPa, and relative elasticity varied between 18 and 25 GPa (Fig. 4e,f,i,j). Surprisingly the hardness of individual KCl crystals on the glue plaque was between 2.0 and 2.1 GPa, and their relative elasticity was 28 to 30 GPa (green arrowheads indicate indents in the substrate, where by deeper indents are indicated by white arrows) suggesting that the KCl crystals are softer than the glue plaque (Fig. 4g,h,i,j). The method of selection of different areas for nanoindentation measurements was exemplified as shown in Fig. 4k, whereas Fig. 4l illustrates displacement nanoindentation curves that were obtained from iterated measurements of a single specific area.

### Adherence of Sgs-glue to different materials (pull-off force or tension)

Bioadhesives are usually high molecular weight, biocompatible, biodegradable polymers used to join two surfaces, where at least one of them can be a living organism. Therefore, it is of general interest to understand the mechanism of natural glue adherence to different materials, this will provide inspirative and essential data for the development of new biomimetic derivatives. After we had initiated our experiments, Borne et al.^35^ published a paper on the universal adhesion of Sgs-glue to different substrates. We found that data collected in the early stages of our experiments supported strikingly different conclusions. As described below, surface area, type of material, hydrophobicity, and electrical charge all influence glue adherence. Therefore, we obtained additional data that would allow us to place glue adherence in the context of the above-described observations of the solidified glue plaque. Altogether we tested 83 different materials and 85 different surfaces that can be grouped into eight categories. More than 5000 puparia were studied during a period of almost 4 years. As discussed below, our division of materials into eight categories ([**A**] to [**H**]) is somewhat artificial but allows for an easier presentation of the data.

As mentioned above, when we sought to remove the freshly formed puparium from the substrate to perform microscopical observations on the Sgs-plaque, an animal had to be removed within the first 10 to 20 s after glue solidification to avoid causing serious injury. However, in the course of numerous and repeated experiments, we discovered that if there was a long gap [ideally more than 15 h after puparium formation (APF)] between tanning and sclerotization of the puparial case and removal of the animal from the glass slide, animals were less vulnerable and even resistant to injury, and could even be used for mechanical/tension measurements. To be cautious and maintain standard conditions, animals 24 to 36 h APF were used for all subsequent measurements. Under these conditions, no animals were damaged during over 5000 detachment experiments.

**Figure 5:**
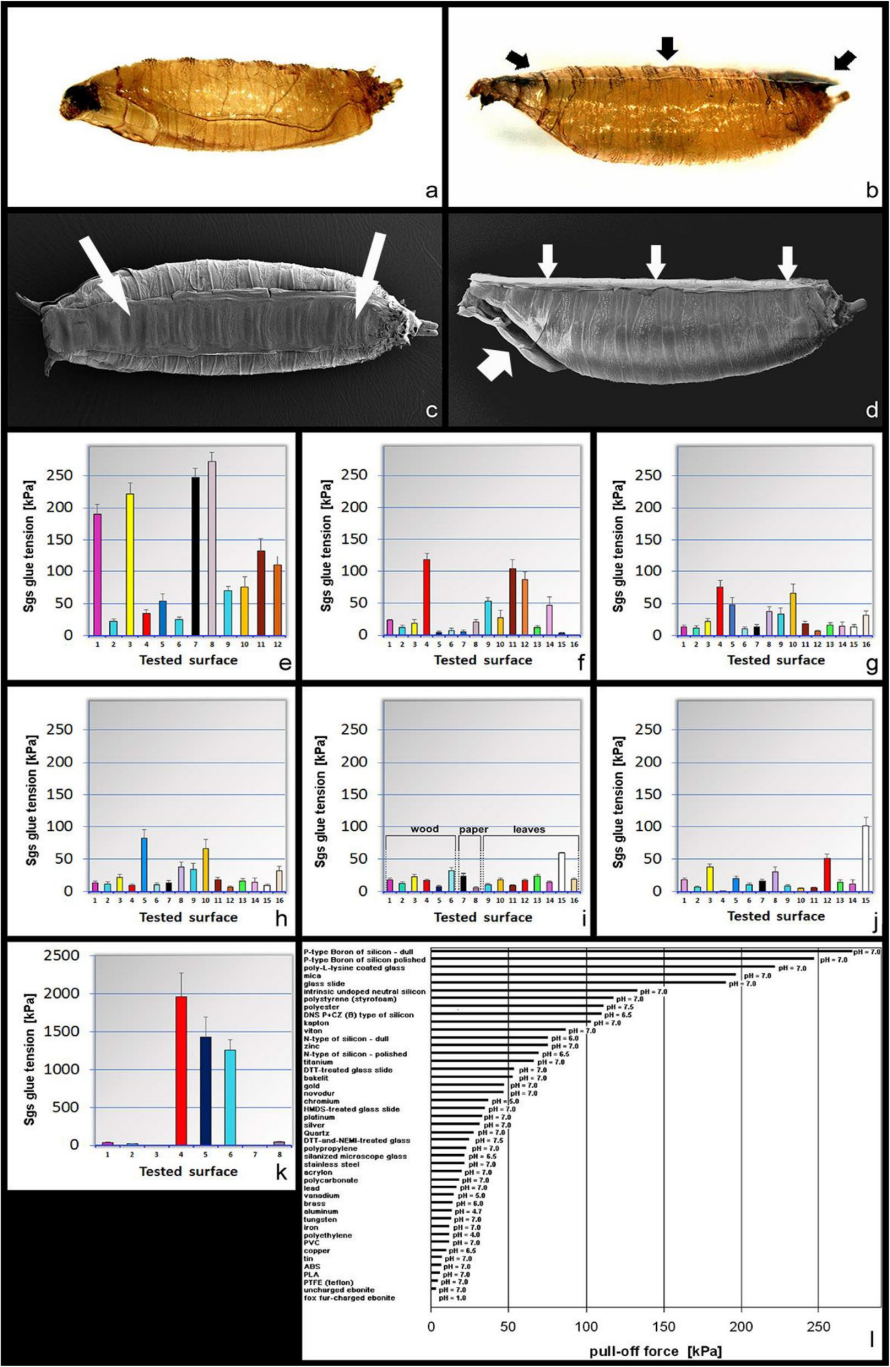
Adhesion of Sgs-glue to different materials (tension measurements) as the pull-off force necessary for puparium detachment (expressed in kPa). (**a**) The puparium removed (detached) from a favorable surface had no Sgs-glue on its ventral side. The Sgs-glue plaque remained on the adhered material leaving there a fingerprint, as shown in Fig. 1. (**b**) Puparium removed (detached) from an unfavorable surface had a clearly visible narrow layer of transparent glue (black arrows) on its ventral side. (**c**) Bottom site of the puparium with adhered Sgs-glue plaque viewed under SEM (large white arrows). (**d**) Side view of the puparium with the adhered Sgs-glue plaque on its ventral side (three white arrows) seen under SEM. Thicker white arrow on the left indicates opened operculum after fly eclosion. In (**a**) to (**d**) the anterior is to the left, posterior to the right). (**e**) Values of Sgs pull-off force on various glasses (treated and untreated) and related materials as silicons. Note that P-type Boron silicons, clear untreated, and poly-L-lysine treated glasses displayed the strongest adherence with Sgs-glue. 1 = soda-lime microscope glass slide, 2 = silanized soda-lime microscope glass slide, 3 = poly-L-lysine coated microscope glass slide, 4 = hexamethyldisilazane-coated microscope glass slide, 5 = Dithiothreitol (DTT)-treated microscope glass slide, 6 = DTT + *N*-ethylmaleimide-treated microscope glass slide, 7 = polished side of P-type boron silicon, 8 = rough side of P-type boron silicon, 9 = polished side of N-type silicon, 10 = rough side of N-type silicon, 11 = intrinsic undoped neutral silicon, 12 = DNS P + CZ (B) type of silicon. (**f**) Pull-off force of Sgs-glue on various plastic materials among which clear hard polystyrene, kapton, and viton had the highest adherence, albeit much less than any glass. 1 = polypropylene, 2 = polyethylene, 3 = polycarbonate, 4 = clear hard polystyrene, 5 = teflon (polytetrafluoroethylene, PTFE), 6 = acrylonitrile butadiene styrene (ABS), 7 = polylactic acid (PLA), 8 = acrylon (polymethylmetacrylate plexiglass), 9 = bakelite (polyoxybenzylmethylen-glycolanhydride), 10 = polyurethane, 11 = kapton, 12 = viton, 13 = polyvinylchloride (PVC), 14 = novodur (hardened PVC), 15 = uncharged ebonite, 16 = fox fur-charged ebonite. (**g**) Sgs-glue pull-off force for various metals, among which zinc and titanium showed the highest values whereas copper and tin had the lowest ones. 1 = aluminum, 2 = iron, 3 = stainless steel, 4 = zinc, 5 = gold, 6 = copper, 7 = brass, 8 = chromium, 9 = platinum, 10 = titanium, 11 = nickel, 12 = tin, 13 = lead, 14 = vanadium, 15 = tungsten, 16 = silver. (**h**) A great majority of woods were unfavorable materials for Sgs-glue adhesion, however, oak and fir provided the best values among them. 1 = common spruce, 2 = pine tree, 3 = maple, 4 = common beech, 5 = oak, 6 = alder tree, 7 = cherry birch, 8 = lime tree, 9 = cherry tree, 10 = fir tree, 11 = apple tree, 12 = pear tree, 13 = apricot tree, 14 = peach tree, 15 = plum tree, 16 = European ash. (**i**) The rest of the wood samples, papers, and plant leaves were poor substrates for Sgs-glue; maple leaf was a slightly better substrate than others, but still not a favorable one. 1 = plane tree, 2 = alder tree, 3 = larch tree, 4 = bamboo, 5 = mahogany wood, 6 = walnut tree, 7 = office paper, 8 = filter paper, 9 = apple leaf, 10 = dandelion (blowball) leaf, 11 = linden leaf, 12 = plantain (ribwort) leaf, 13 = acacia leaf, 14 = poplar leaf, 15 = maple leaf, 16 = raspberry canes leaf. (**j**) Among stones, rocks, and minerals the clear favorite for Sgs-adhesion was positively charged mica; marble was on the opposite end. 1 = granite, 2 = quartz, 3 = aragonite, 4 = marble, 5 = gypsum, 6 = chalcedony, 7 = serpentinite, 8 = opal, 9 = clastic conglomerate, 10 = dark-red agate, 11 = green agate, 12 = blue agate, 13 = ammonium alum, 14 = shale, 15 = mica muscovite. (**k**) Both graphenes, as well as ceramic tiles were relatively weak surfaces in comparison to glass, silicon, and notably chitins. The chitins were clearly ideal surfaces for Sgs-glue adhesion even in the case of cuticles from heterologous species, such as *Tenebrio molitor* and *Galleria mellonella* (bars 4 and 5, respectively). 1 = graphene oxid, 2 = acid-reduced graphene, 3 = chitin (*Drosophila* puparial case), 4 = chitin (*Tenebrio* adult elytron), 5 = chitin (*Galleria* pupal case), 6 = ceramic tile. (**l**) A list of the first 45 materials used in our adhesion study that were ordered from most favorable (top) to least favorable (bottom) for glue adherence. Generating this ordered list revealed that many list members and their relative position correspond to a triboelectric series known from physical and material studies (for a more detailed comparison see "Discussion").

We found that there are two major groups of surfaces based on the strength of their interaction with the Sgs-glue: (1) compatible surfaces on which the solidified Sgs-glue stays attached after mechanical removal of the puparium, and (2) incompatible surfaces from which the solidified Sgs-glue is taken off with the puparium upon its mechanical removal, and stays attached to the puparium in the form of a transparent foil (Fig. 5a,b). Due to this fact, the strength, expressed in kiloPascals (kPa), necessary to remove the puparium from the substrate, had to be calculated according to the real size of the area from which the puparium was removed. Under group (1), this area corresponds to the area of the puparial fingerprint in the glue, while under group (2), it corresponds to the entire area of the solidified glue. To perform calculations, these areas were photographed and measured under the microscope for each individual experiment, and the area was calculated as described in detail under Materials and Methods (see also Fig. 2). In the case of solid non-transparent materials, microscope images of the glue had to be taken under incident light illumination. Only wild-type (non-GFP) animals were used throughout this part of the study. Furthermore, it should be noted that in group 2 (incompatible surfaces) the Sgs-plaque is made of a single smooth layer of the glue where individual cascading terraces are usually less apparent or even absent, because animals on these surfaces, notably PTFE and bibulous paper, are unable to perform fully functional semicircular movements with their head to spread the glue properly.

Fruitflies are traditionally reared in glass vials for hundreds of generations, and the larvae use their glass wall for cementing the puparia. Thus, we selected microscope slides made of soda-lime glass, additional glass-related materials, and materials with purposely modified surfaces with regard to the electric charge as our first materials to test the adhesion of Sgs-glue. Then, in the next series of experiments, we tested different materials that were easily available to us, including various plastics, metals, fabrics, and natural materials such as stones and minerals, plant leaves, papers, and the wood of different trees.

The average value of puparium pull-off force on the clean untreated microscope glass slide was 189.76 kPa, whereas silanized microscope glass required 21.36 kPa, poly-L-lysine coated glass slide required 211.45 kPa, HMDS-treated glass slide required 34.28 kPa, DTT-treated glass slide required 53.12 kPa, and combined DTT-and-*N*EMI-treated glass required 24.17 kPa. A closely related group of materials are various types of silicon, for which we also measured the following pull-off force values: polished side of a P-type Boron silicon wafer (8–12 Ω cm) 226.61 kPa, rough side of P-boron silicon wafer (8–12 Ω cm) 241.77 kPa, polished side of N-type of a silicon wafer (10–12 Ω cm) 69.14 kPa, rough side of N-type of a silicon wafer (10–12 Ω cm) 75.28 kPa, intrinsic/undoped neutral silicon wafer (> 10,000 Ω cm) 132.6 kPa, and DNS P + CZ (B) type of silicon wafer (0.005 to 0.015 Ω/cm) 109.71 kPa (Fig. 5e).

Based on these results, we were encouraged to extend our observations to a variety of different materials including plastics, metals, woods, paper, plant leaves, stones and minerals, graphene, and natural chitins. For this study, the initial selection of materials was random, mostly based on their availability, but after obtaining the first results and forming a general understanding of the adhesive properties of the glue to different materials, we targeted additional materials based on their specific properties.

The majority of plastics were very poor substrates for the adhesion of Sgs-glue (exemplified by PTFE, PLA, ABS, and ebonite) (Fig. 5f). Surprisingly, clear polystyrene, kapton, and viton were unexpectedly acceptable materials for Sgs adhesion, although not as good as glass. Novodur (non-softened polyvinylchloride) and bakelite (polyoxybenzyl-methylen-glycol anhydride, which is one of the thermosetting phenol formaldehyde resins) produced middle-range adhesivity values among plastic materials (Fig. 5f). In contrast, classical polyvinylchloride gave fourfold less adhesiveness, and the major difference between them is the absence of phthalates in novodur. Ebonite rubbed against fox fur, which makes it strongly negatively charged did not allow any larva to stay on its surface and prevented any adhesion, making it less acceptable to Sgs than PTFE.

In general, the great majority of metals, if compared to glass and silicons or polystyrene and kapton, are poor substrates for Sgs-glue. Among them, zinc, gold, and titanium proved to be better substrates (range close to or above 50 kPa) than others such as iron, copper, or tin (close to or below 10 kPa). The remainder of the tested metals (steel, aluminum, brass, chromium, platinum, nickel, lead, tungsten, and silver) fell into mid-range metallic substrates with adhesiveness values between 10 and 35 kPa (Fig. 5g).

Even though wood, paper, and leaves are natural materials, their adhesion with Sgs-glue was poorer than expected. Except for a few types of wood such as oak and fir which provided an unusually good substrate for Sgs-adhesion (60 to 80 kPa), the majority were found to be poor substrates with values close to or below 30 kPa (Fig. 5h,i). Interestingly, two tested papers (classical office paper and filter paper) were found to be poor substrates. However, it should be noted that there is a considerable difference in surface topography between the two: white office paper is smooth, whereas filter paper is highly porous and displayed more than fourfold weaker adherence (5.7 kPa) for Sgs-glue (Fig. 5i). We also tested bibulous paper, but the presence of cellulose „hairs “ on its surface completely prevented cementing of the puparia, and very often pupariation itself, and therefore no values for the pull-off force could be measured. Furthermore, wandering larvae showed difficulties in moving on this type of surface and were unable to move a few millimeters. Without finding minimum continuous contact with the substrate, they remained as larvae even after 24 h. In the case that they randomly found another larva, they were able to initiate pupariation within 15 to 30 min, accompanied by expectoration of the Sgs-glue which cemented both puparia together. If more than two larvae were lucky to find each other, a group of 3 to 32 puparia was found on the surface of bibulous paper sticking to each other (Supplementary Fig. 8a–c). Importantly, they never stuck to bibulous paper and were freely laying on its hairy surface.

Additionally, we tested 8 different freshly-collected plant leaves among which maple leaf showed the highest affinity to Sgs-glue (58 kPa), and linden leaf the lowest (8.8 kPa). The rest of the plant leaves (dandelion, plantain, apple, acacia, poplar, and raspberry cane) displayed values between 10 and 18 kPa (Fig. 5i).

Among stones and minerals, mica muscovite showed the highest affinity (strong adhesion) to Sgs-glue reaching 100 kPa. Blue agate gave about 50 kPa, aragonite over 35 kPa, and opal close to 30 kPa (Fig. 5j). The rest of the tested rocks and minerals (granite, quartz, gypsum, chalcedony, serpentinite, opal, clastic conglomerate, ammonium alum, and shale) displayed average to low adhesion values ranging from 4 to 20 kPa, including dark-red and green agates. The lowest value was found for marble at below 1 kPa (Fig. 5j).

Because of its availability to us, we also tested two forms of graphene (graphene oxide which is hydrophilic, and acid-reduced graphene which is hydrophobic); they produced pull-off adhesion forces of 37.8 kPa and 15.8 kPa, respectively (Fig. 5k). We also had the opportunity to test the polished (upper) side of ceramic tiles made of potter’s clay which resulted in a pull-off force of 41 kPa that is comparable, for example, to novodur plastic, chromium, gold or lime tree wood (Fig. 5k). The most interesting data came, however, from measuring chitin, a fully natural insect material. It was the specific behavior of larvae on incompatible surfaces such as bibulous paper or PTFE that brought our attention to test pull-off forces on chitin. When individual larvae are left on bibulous paper or PTFE they showed difficulties in their wandering behavior (they moved much less) and tended to delay pupariation significantly. After they pupariated, they would often not reach the pupal stage. However, if the density of larvae on these substrates was increased, they would find each other, and in less than 30 min the majority of them pupariated, and continued pupal development as well as pupal-adult transformation. When two or more (easily 10 or 12) larvae purposely touched each other, they became cemented together by Sgs-glue. We found it extremely difficult to separate these multi-puparia using forceps. Therefore, we decided to place a small number of wandering larvae on PTFE inside small (Ø 5 cm) glass Petri dishes to increase the chance of finding only two animals glued together. From these „twins “, we selected only those which were attached to each other along their entire ventral area (excluding those that shared a little area or were attached by lateral regions). We then fixed one of them to a microscope glass slide with instant superglue (bottom puparium), while the other one was used to mount on its dorsal side the Bossard zinc-chromium coated steel O-ring circle, again with instant superglue (upper puparium). Then, the pull-off force was measured in the usual way as described above. As shown in Fig. 5k (bar 3), chitin required as much as 2 MPa to separate two glued puparia from each other.

Similar experiments were performed with two heterologous sources of chitin: pupal exuvial cuticle of *Galleria mellonella* and elytra of *Tenebrio molitor* adults. Wandering third instar larvae of *Drosophila* were placed in a glass Petri dish lined with PTFE on which exuvial cuticles of *Galleria mellonella* pupae or entire adult elytron of dead *Tenebrio molitor* beetles were randomly scattered. For measurements, we selected only those *Drosophila* puparia which were attached to the pupal cuticle of the *Galleria* or elytron of *Tenebrio* by the entire ventral area. Then, the pupal cuticle or elytron was fixed to the microscope glass slide with instant superglue, while on the dorsal side of the puparium was attached the Bossard zinc-chromium coated steel O-ring circle using instant superglue. Subsequently, the pull-off force was measured as described above. As shown in Fig. 5k (bars 4 and 5), even heterologous chitin required 1.2 to 1.4 MPa to separate glued puparium from the cuticle of different origin.

### Bifurcated glue channels (bidentium)—an anatomical mystery in *Drosophila*

**Figure 6:**
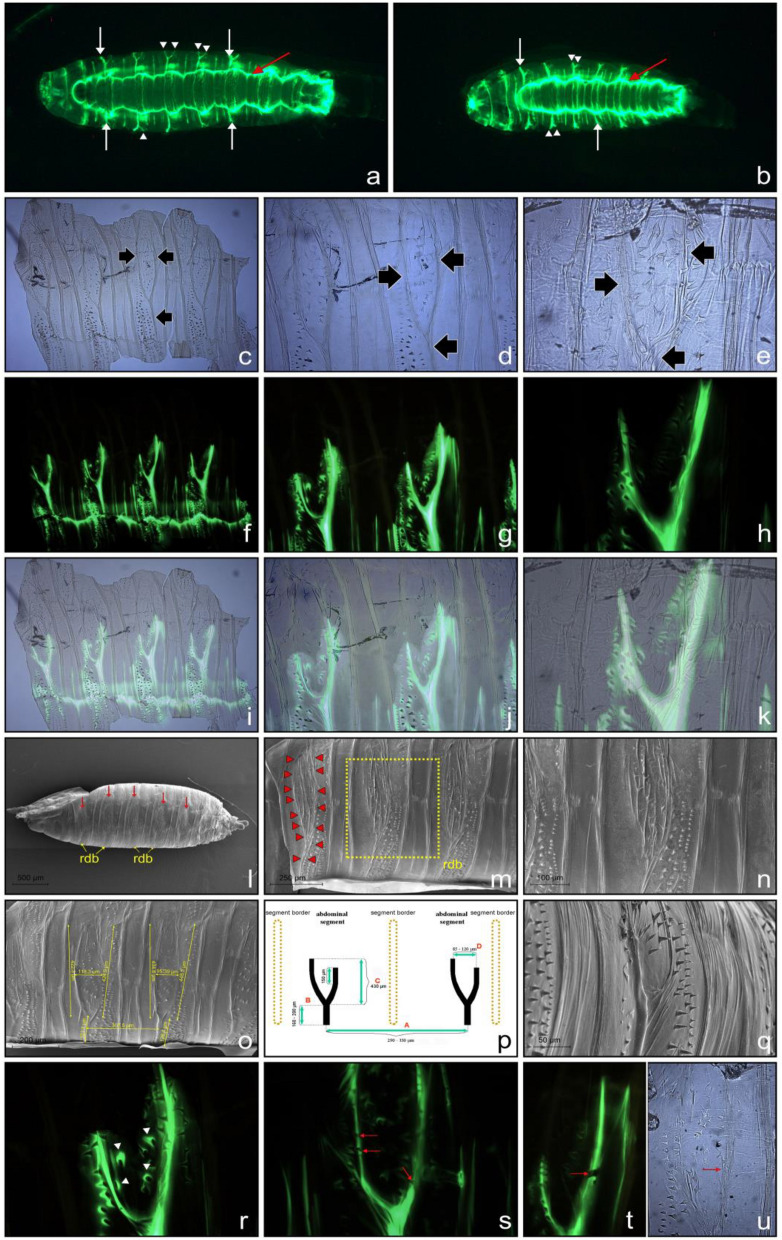
Bidentia—bifurcated glue channels on the cuticular surface of puparial abdominal segments. (**a**) and (**b**) Fluorescence micrographs of Sgs3-GFP signal in two independent freshly formed puparia glued to the microscope glass slides; images were taken from the bottom side. White arrows point to the channels where Sgs-glue was streaming dorsally up on the ruffled cuticle of abdominal segments. White arrowheads point to the upper part of the bifurcation on the lateral segments. The red arrow indicates the circumference of the fingerprint area, whereas yellow arrowheads point to the ventral denticle belts. (**c**) to (**e**) Bright field microscopy view of cuticular preparations from the lateral side of abdominal segments showing bifurcated Y-like structures (black arrows) at two different magnifications. (**f**) to (**h**) Images of the same portions of cuticles as in (**c**) to (**e**) but under fluorescence illumination providing evidence that the Y-like structures corresponded to the Sgs3-GFP signal observed under low magnification whole-mount preparations. (**i**) to (**k**) document the overlap of Y-like structures from (**c**) to (**e**) images with the GFP signal shown in (**f**) to (**h**). (**l**) to (**n**) SEM images of the puparial case (after emergence of the fly) showing the presence of bidentia. (**l**) Whole view of an empty puparium where bidentia are indicated by red arrows; yellow arrows point to ventral denticle belts (vdb). (**m**) Higher magnification of a bidentium on the 3^rd^ abdominal segment, which is delineated by a series of red arrowheads. At the bottom of the puparium, a thin layer of solidified Sgs-glue is visible. „vdb “ indicates ventral denticle belts. The yellow rectangular frame demarcates the area of a single bidentium viewed at higher magnification in (**n**). (**o**) Measurement of individual parameters of the bidentia directly on lateral abdominal segments of the puparial case, showing that the main trunks are cca 300 µm apart (distance red **A** in (**p**)), the length of upward oriented lateral trunks is around 400 µm (distance red **C** in (**p**)), and the distance between them within a single individual bidentium is 85 to 120 µm (distance red **D** in (**p**)). (**p**) Schematic drawing of a pair of bidentia based on more than 100 individual measurements. The numbers shown are ranges reflecting the median of these measurements. The height of the main trunks (red **B**) is higher than what can be shown in a single SEM image, as it required at least two or three tilting movements of the microscope sample holder to see their entire length. In addition, in animals where solidified Sgs-glue is cemented to the ventral side of the puparium, a significant portion of the main trunks is hidden by this glue. (**q**) The side view of the lateral segments shows a relatively complex topography of the cuticle and reveals that trunks and cacumens (tips) of these Y-like structures are concave and extend above the surrounding cuticle area resembling diverticula. (**r**) Detailed image of a GFP-Sgs bidentium obtained at higher magnification documents that additional fluorescent signal spreads inside and outside the Y-shaped areas (white arrowheads). (**s**) and (**t**) Interrupted GFP-signal due to small local cracks of otherwise continuous Y-shaped bidentia (red arrows). To evenly spread cuticular preparations in mounting medium, we used a polished brass weight cca 20 g (15 mm in diameter) that we placed gently on the coverslip. As shown in (**u**) this helped to spread the chitinous cuticle without any disturbance or interruption to it (red arrow), but it could cause occasional cracks in the GFP-labeled Sgs-glue shown in (**s**) and (**t**). This indicated that the GFP-labeled Sgs spreaded on the surface of the Y-shaped integument, and that bidentia are not cavities or capillaries. It also indicated that the cuticle was more elastic than Sgs-glue.

The use of the *Sgs3*-*GFP* transgenic construct not only allowed us to observe under fluorescence the dynamics of glue release, but also to investigate the variability in the gross morphology of the glue plaque and its distribution (see Fig. 1f). A closer look at the distribution of the fluorescent GFP signal revealed that in addition to the main plaque and fingerprint, there is a clear and repeated pattern of lateral canaliculi via which the Sgs had streamed upwards dorsally on the ruffled cuticle of the abdominal segments (Fig. 6a,b). Although there was individual variability among animals regarding the distance the glue traveled, the GFP signal always streamed in the ventral-to-dorsal direction, and usually bifurcated between a third to a half of the way from the ventral side of the puparium.

This prompted us to investigate the regular segmental pattern of Sgs-distribution more closely. Primarily we found similar structures on permanent preparations of lateral cuticular segments from puparia (Fig. 6c–e). Examining Sgs3-GFP-labeled cuticles under a fluorescence microscope revealed that these structures and GFP signals fully overlap (Fig. 6f,g,i,j,k). On some preparations, the anterior branches of these bifurcated GFP-signals on each segment appeared longer than the posterior, whereas in the majority of others, it is vice versa, but the direction of the assymetry is always uniform in one animal (compare Fig. 6f,g,h). To obtain more detailed data on these novel two-pronged Y-like structures, we used SEM. As illustrated in Fig. 6l–n, previously unattended structures were present on every lateral segment of the abdomen (usually distanced 290 to 330 µm from each other and with trunks 110 to 180 µm tall). Bifurcated branches were variable in length (350–450 µm). The average distance between these branches was 80 to 120 µm (see Fig. 6o,p). The distribution of bidentia sizes/distances is depicted in histograms in Supplemental Fig. 3. In contrast to the original images obtained using a fluorescence stereomicroscope (e.g. Figs. 1d, 6a,b), both SEM and fluorescence microscope images document that these novel structures are more complicated and show multiple smaller cuticular ramifications. A partial side view of the lateral segments shows the relatively complex topography of the cuticle, and reveals that trunks and cacumens (tips) of these Y-like structures are concave and extend above the surrounding cuticle area (Fig. 6q). Moreover, their detailed pictures obtained at higher magnification under fluorescent light clearly documents low-level signal spread inside the Y-shaped areas (Fig. 6r). Most importantly, when mounting these cuticles under a cover slip (regardless of their gentle handling and the protocol applied), the continuous GFP-signal was interrupted in a few locations due to small local cracks above the cuticular Y-shaped structures (Fig. 6s,t). These places strongly resemble cracks in dried paint that has become loose from more strongly adhering plaster of a wall. This provides the clearest evidence that the GFP-labeled Sgs spreads on the surface of the integument, and that the puparial Y-shaped structures lack cavities and are not capillaries. Since we were not able to find this or any similar-looking structure described in the literature, we propose that it be considered as a new structural element. We coined the name „bidentium “ (plural „bidentia “) due to its resemblance to a bident.

**Figure 7:**
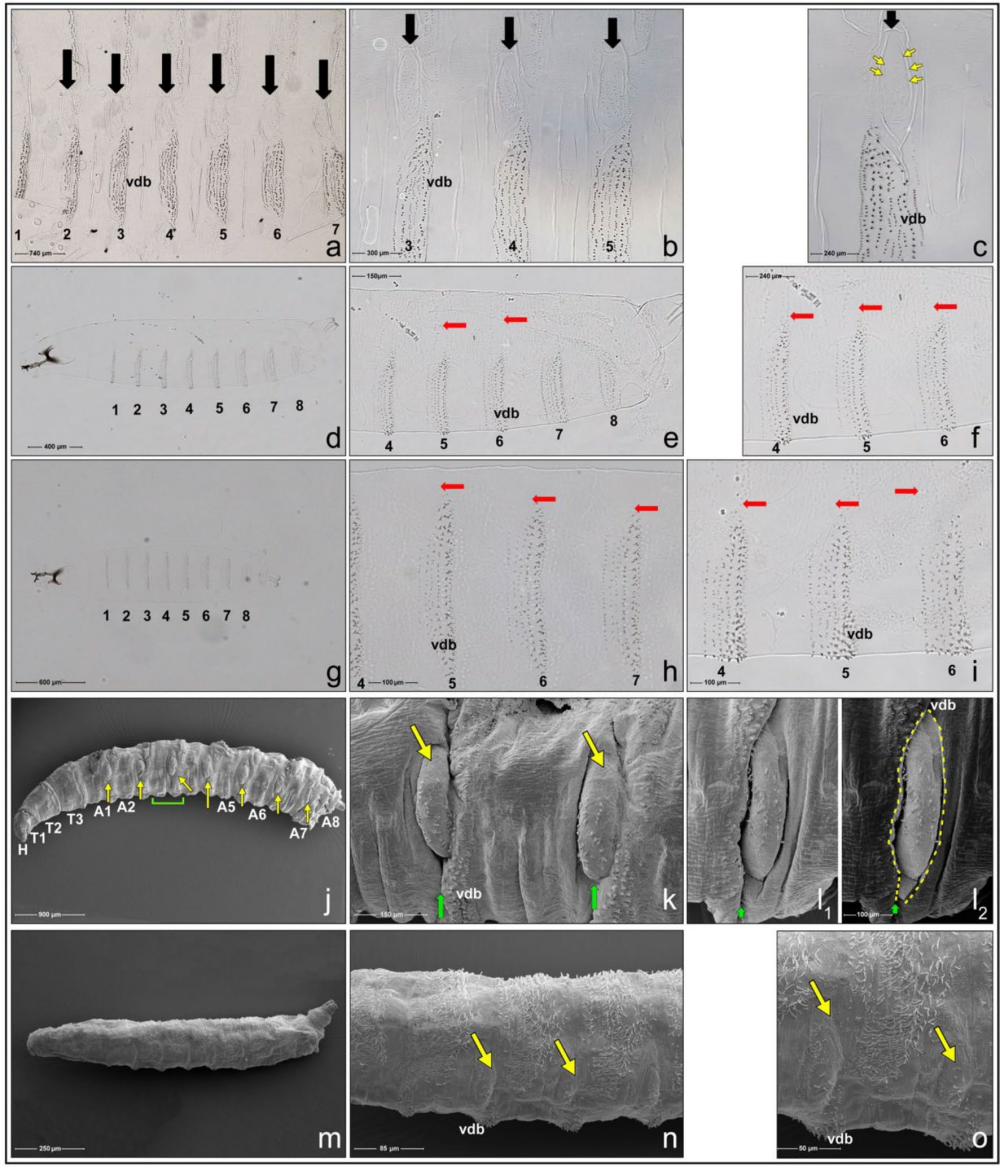
Bidentia in larval stages as seen in light and scanning electron microscopy. (**a**) to (**c**) Bidentia (black arrows) are present and clearly visible in cuticular preparations of 3rd instar larvae, stemming from the anterior region of ventral denticle belts (vdb); numbers indicate individual abdominal segments. In 2nd instar larvae ((**d**) to (**f**)) and 1st instar larvae ((**g**) to (**i**)) these structures are absent (red arrows) even if viewed under higher magnification. In these cuticular preparations mounted in Hoyer’s medium bidentia in 3rd instar larvae appear like local chitinous diverticula nested in the smooth cuticle. Inside bidentia are present 18 to 23 individual hairs (yellow arrowheads in (**c**)). However, bidentia, including hairs in appropriate areas, are completely absent in larvae of 2nd and 1st instars (red arrows). Identification of bidentia using SEM in 3rd instar larvae was facilitated by the fact that they form local invaginations around epaulette-like tubercles on each abdominal segment (**j**). Although bidentia could be recognized, they were not as apparent as on the puparial case (compare Fig. 6). Bidentia appeared as invaginations or impressions surrounding tubercles. (**k**) Two tubercles (yellow arrows) on 3rd instar larval abdominal segments surrounded by invaginated cuticle, which represent a bidentium. Short green arrows indicate the direction of the flow of Sgs-glue. (**l**) Detailed view of a single tubercle with bidentium demarcated by a yellow freeform line, which was purposely shaped to indicate the maximum height which the glue can stream up. A short green arrow points to the direction of the flow of Sgs-glue. (**m** and **n**) Tubercular structures (yellow arrows) could be recognized on the lateral side of abdominal segments of the 2nd or 1st instar larvae, but there were no indications for bidentia (**n** and **o**, respectively). vdb = ventral denticle belts. These observations also indicated that the segmental tubercles visible under SEM were not easily detectable in whole-mount cuticular preparations, even during the 3rd larval instar, and therefore a combination of these two techniques had to be used for the unambiguous identification of bidentia.

We stress that while bidential structures are particularly apparent on the puparial case (see Fig. 6c,d,e,k,l,m,n), they are also relatively visible in late as well as early-to-mid third-instar larvae. We documented this using light microscopy (Fig. 7a–c). However, a different picture can be seen via the use of SEM. As illustrated in Fig. 7j,k,l, third-instar larval bidentia appear as a sort of invaginations or impressions surrounding tubercles. Except for the distance between individual bidentia which is longer in stretched larvae due to unshrunk segments, all other dimensions associated with bidentia are in the same range as those described above for puparia. It is reasonable to suggest that the visibility of bidentia in puparia is profoundly increased by abdominal peristalsis during pupariation, followed by puparial case hardening and melanization. Strikingly, we also emphasize that the peristaltic movements are performed by pupariating larvae at a time when bidentia are invaginated, so this aids or facilitates the flow of the liquid glue via the local impressions they provide. Then the Sgs-glue solidifies quickly, and the bidentia in puparia become concave after the glue has passed through such invaginations.

After several repeated trials, we were unable to identify any bidentia in the first- or second-instar larvae (see Fig. 7d–i,m–o). This indicates that the formation of bidentia is strictly associated with the transition from second- to third-larval instars, and reflects the developmental commitment of the animal to undergo metamorphosis. Furthermore, along with mandibular hooks, anterior and posterior spiracles, tracheal trunks, caudal sense organs, and dorsal spinule raws, that all continuously develop from the embryonic period and provide typical characters for each instar, bidentia can serve as a unique and distinctive morphogenetic feature of the last larval stage.

## Discussion

### Summary of key findings

This work describes key aspects of how the pupariating larva becomes cemented to the substrate. In many descriptions^35–44^, it is the pupa that becomes cemented to the substrate. This imprecise description conveys developmental, morphological, functional, and ecological misconceptions, however. From a developmental perspective, it is the mature larva that releases the cementing glue to affix itself to the substrate. Temporally this occurs after the larva becomes almost immobile. Only afterwards is pupariation completed to form the prepupa^45^. From a morphological perspective, the prepupa forms from the larval exuvia during a stage lasting about 13 h. Afterwards, formation of the pupae requires successful apolysis, synthesis of new pupal cuticle, expelling of the mouth hook armature, and retraction of the head capsule^46,47^. From a functional perspective, pupariation of the larvae is linked to development, but not to substrate attachment. A larva can pupariate and become fixed to a substrate by glue from the salivary glands, but if pupariation is stopped during the prepupal stage due to an exogenous disruption or mutation, further development stops and the animal will not pupate, even though it remains glued to the substrate. Indeed, while the pupa forms inside the puparial case, it is formed much later, and when it forms, the pupal cuticle lacks contact with its outside puparial case. The pupa lacks the larval salivary glands that produce the Sgs-glue: they were completely histolyzed by programmed cell death in the early pupal stage^6,30,48^. Successful Sgs-glue secretion by the larva plays an additional developmental role: our own observations indicate that successful glue secretion and attachment of the prepupa to the substrate may be required for accomplishing pupation and subsequent continuation of pupal-to-pharate adult transformation. Finally, from an ecological perspective, it is the larva, and not the pupa, that seeks and chooses the preferred pupation site. Thus, it is imperative to convey that the larval exoskeleton that is retained by the prepupa, which becomes the puparial (or prepupal) case, but not the pupa, becomes glued to the substrate.

This work provides more detailed insights into the gross-morphological appearance of the secreted and solidified glue than have been available to date. Our observations of pre-prepuparial behavior of third-instar larvae of *D*. *melanogaster* during glue expectoration are very similar to those described by Fraenkel and Brookes^12^ in *Phormia regina*. Very recently, Heredia et al.^49^ studying the function of the *Dilp8*-*Lgr3* signaling pathway obtained a high-resolution video of this process employing the same *Sgs3*-*GFP* fusion construct we used in this study. Congruent with our observations, they demonstrated that most of the viscoelastic Sgs-glue is released during 30–60 s of intense abdominal peristalsis that squeezes the glands to expectorate the glue. Importantly, this facilitates its efficient spreading beneath the ventral cuticular segments and along the entire anterior–posterior axis. When the abdominal peristalsis stops, there are still visible remnants of the glue inside the SG lumen. We add to these findings here, showing that at this moment, when the body has become immobile and starts to sclerotize, the head makes semicircular movements around the thorax, while the mouth pulsates back and forth for another 20–30 s. These limited movements serve to release the last doses of remaining glue from the lumen near the head and thorax. This leads to the glue forming a plaque with alluvial-like cascades or terraces (Fig. 1g–l). Thus, cementing the puparium to the substrate is at least a two-phase process: (1) the release of the majority of the glue via abdominal peristalsis, which leads to the formation of the fingerprint, and (2) the release of the remaining glue during semicircular head movements, which leads to the formation of a plaque having alluvial-like cascades. Both act in concert under the genetic control of the *Dilp8*-*Lgr3* signaling pathway.

### Gross-morphological properties of the solidified glue

Previously we described the surface and internal substructure of the expectorated and solidified Sgs-secretion^33^. We found an internal spongious-to-trabecular infrastructure that reflects the state of glue hydration with „fibers “ with highly irregular thicknessnes. This study demonstrates that the cured glue has a highly complex topology. The watery fluid comprising the expectorated glue solidifies into a harded cement within seconds and has two distinct elements: an enamel-like plaque composed of a central fingerprint and more laterally located layers of cascading terraces. The fingerprint reflects the features of the ventral exoskeleton of the puparial case (the former larval cuticle), and mirrors the head, thoracic and abdominal segments, including their borders and denticle belts (Fig. 1g–j). This central fingerprint is surrounded by 3 to 12 layers of cascading terraces that form during the back-and-forth pulsations of the mouth. Borne et al.^35^, recently described the fingerprint as the „glue-substrate interface “, but failed to describe the alluvial-like cascading terraces perhaps because described only lateral views of the solidified glue using Z-section projections of the cuticule-substrate interface. Our findings reveal that the substrate attachment mediated by the glue is significantly fortified by the addition of the terraced alluvial-like cascades. Since the single-layered block of cement comprising the fingerprinted area can contain gaps between the puparial case and the substrate, adding an assemblage of multilayered terraces provides adhesion whose elasticity makes it more difficult to detach. This provides attachment stability if environmental conditions become adverse before eclosion.

### Air-accessible surfaces of the solidified glue are pocked with KCl crystals

We also report that numerous small „grains “ are restricted to the alluvial-like cascading terraces; they are never observed within or nearby the fingerprints (Fig. 1i–l). Further investigation of these previously undescribed elements through AFM, SEM, and three different diffractometric and mass-spectrometric analyses revealed that they are relatively large, 0.1-to-1 µm crystal-like formations predominantly containing potassium chloride (KCl) (Fig. 3a to 3p). The unavailability of oxygen within and nearby the fingerprints—where there is direct contact of glue to both the substrate and cuticular exoskeleton—may explain their presence only within the terraced regions.

Crystallization is a typically a slow, multistep process having two major phases: nucleation and growth. It has several subphases which may take hours-to-weeks; in geological time, it can be years or longer. Since the watery expectorated glue solidifies in seconds, the KCl-containing crystals must form within seconds. Hence, their formation on oxygen-accessible surfaces of the Sgs-plaque is extraordinarily fast. This extraordinary dynamic suggests that crystal formation is driven catalytically. The major phases of crystallization—nucleation and growth must be present, however. The hundreds to thousands of crystals suggest multiple nucleation sites, and the different sizes indicate growth. It is generally accepted that a smaller ratio of surface area to volume in larger crystals can lead to higher purity^50–52^. Our energy dispersive spectroscopy, electron backscattered diffraction, and secondary ion mass spectrometry analyses showed that the crystals were composed of relatively pure KCl with few minority components.

Physical factors that could affect the rate of KCl crystallization from the expectorated liquid glue are limited. Crystallization per se is a phase transition, so factors such as cooling (temperature) and changing the solvent mixture composition are excluded. We suggest that rapid crystal formation is facilitated by an evaporative type of crystallization accompanied by reasonable velocities of exchange of surface modalities. The observed range of crystal sizes is consistent with this hypothesis, since evaporative crystallization tends to yield a larger average-crystal size and narrows the crystal-size distribution curve. In contrast, laboratory or industrial crystallization, or slow crystallization in nature, usually yields a much broader distribution of crystal sizes^53^. Crystallization results from the physical transformation of a liquid to a crystal, a solid with an ordered internal arrangement of molecules, ions, or atoms^54–60^. We refer to the KCl aggregates on the glue as crystals even though repeated X-ray diffraction analyses failed to confirm their exact internal structure. This is because, if any crystal-like structures arose, they must have been started with periodic crystal structure initiation points, and their external appearance reflects their internal crystal structure arrangement. While we observed crystal-like or crystallite solids of KCl, the existence of true periodic crystals remains a possibility.

Since hemolymph has a high concentration of KCl^61–64^, it could be the source of most of the crystallized KCl. A functional hypothesis is that KCl is required prior to expectoration for the maintenance of glue in a watery state, perhaps by regulating solubility or catalytic activity, or for the structural integrity of glue components. If KCl attenuates or inhibits a rate-limiting step in glue maturation while it is within the secretory vesicles or inside the SG lumen, its removal upon the glue’s contact with air (oxygen) by crystallization would facilitate the sudden activatation of glue solidification, or an other process that indirectly leads to the quick formation of a glue cement. An alternative explanation for why KCl crystals appear only on terraces is that a high concentration of KCl arises from a temporally limited activation of fast-acting ion pumps/membrane channels within the SGs, specifically when there are limited mouth movements during the final stages of glue expectoration. This too would support the hypothesis that Sgs-glue release and curing is a multi-step process.

### Mechanical properties of Sgs-glue

After removing the freshly formed puparia, we initially characterized the mechanical properties of the solidified Sgs-plaque terrace, fingerprint, and KCl crystals using nanoindentation to quantify their hardness (H), a measure of a material’s resistance to deformation by surface indentation, and relative elasticity (E_r_, elastic modulus, Young’s modulus), an intrinsic material property that relates deformation to the motion of dislocations in a material’s atomic structure^65–69^. The hardness of the Sgs-plaque terrace was only slightly lower than that of the soda-lime glass on which the Sgs-glue solidified (Fig. 4j). Its relative elasticity was also slightly lower than that of the soda-lime glass. This was some what surprising, as we expected the Sgs-plaque to show higher elasticity than the hardglass. While there is no direct relationship between a material’s elastic modulus and hardness^65,70,71^, it is generally accepted that a material’s hardness tends to increase with its elastic modulus^72,73^. In this sense, Sgs-glue does not differ from numerous synthetic materials.

The fingerprint had significantly lower hardness than the Sgs-plaque. Its relative elasticity was also lower, but not proportionally (Fig. 4i,j). This indicates that the thin and stretchy finger print regions, which may have small zones not directly adhering to the glass slide are more highly flexible and soft.

Probing the hardness of the KCl crystals revealed they were softer than the Sgs-glue plaque. This suggests that the significant hardness of the biocement is achieved by polymerization of glue components, a hypothesis consistent with the speed of solidification of the expectorated glue. Though the KCl crystals were expected to have lower elasticity, they had a surprisingly high E_r_ of 29 to 30 Gpa. This corresponded exactly with the E_r_ reported in crystal databases (KorthKristalle database, MathWeb, Optic-Org, Halide-CryLink). The same databases report hardness values 15 to 23-fold lower than we found (2.1 GPa), however. This substantial difference suggests the fast nucleation and growth of the crystals formed on the Sgs-glue plaque surface results in crystals with different properties than slowly grown crystals.

Since there are very few data on the mechanical properties of bioadhesives, it would be useful to compare the *Drosophila* Sgs-glue to other bioadhesives. We made preliminary observations on adhesive skin secretions of three species of salamanders (*Ambystoma opacum*, *A*. *maculatum*, and *Hynobius dunni*). In a preparation resembling the solidified Sgs-plaques, we collected the milky mucous skin secretions as smears on clean glass slides^74,75^ and used nanoindentation measurements to quantify their hardness and elasticity. The hardness of the secretions for *A*. *opacum*, *A*. *maculatum*, and *H*. *dunni* were all 0.3 ± 0.1 GPa, while their E_r_ were 6.8 ± 1.2 GPa, 5.0 ± 0.5 GPa, and 6.8 ± 1.0 GPa, respectively. It is remarkable that these three species, even though they live on different continents and inhabit different environments, have secretory bioadhesives with similar hardness and elasticity. Furthermore, it is striking that the *Drosophila* Sgs-plaque is over 14-times harder, and displays a 6 to 9-fold higher elasticity. Taken together, these observations suggest that bioadhesive properties are evolutionarily fine-tuned.

### Triboelectric charge plays a pivotal role in glue adhesion

We used a wide range of materials to evaluate the adhesive properties of the Sgs-glue. After initially evaluating materials used successfully for rearing *Drosophila* cultures (soda-lime glass and solid polystyrene), and a glass-related material, silicon, we evaluated other plastics. Because of striking adhesive differences between polystyrene and other plastics, we expanded our evaluation to include metals, and then natural materials (rocks, minerals, woods, and plant leaves). Since *D*. *melanogaster* is not only a cosmopolitan but also a synanthropic species, we also tested synthetic fabrics and materials. Testing a wide range of materials was instrumental in uncovering the crucial factors required for Sgs-glue adhesion and showing that materials were divisible into two groups independent of type, origin, and composition: adhesion-favorable and adhesion-unfavorable materials. Homologous chitin surfaces (*Drosophila* puparium), as well as heterologous chitin surfaces (*T*. *molitor* adult elytrum and *G*. *mellonella* cuticle) were the most favored adhesion substrates. This is expected as chitin is an obligatory substrate for the adherence of the expectorated glue. Among facultative (*i*.*e*., secondary, different from chitin) substrates, the order of groups, relative to the proportion of favorable materials, were glasses and silicates (8 of 12), plastics (3 or 4 of 16), metals (2 of 18), woods (2 of 22), and stones and minerals (1 of 15), with no papers or plant leaves offering favorable surfaces (Fig. 5). When we ordered the list of about 45 facultative substrates by adhesivness to Sgs-glue, the order of materials with known triboelectric charge strongly resembled a triboelectric series (see^76–81^ for a comparison of triboelectric series). This result is in striking contrast to the conclusions of Borne et al.^35^, but they tested only a limited number of materials. We then confirmed our hypothesis about the significance of triboelectric charge by further expanding the number of tested materials (Fig. 5l), almost all of which can be organized as either neutral or able to acquire a positive or negative triboelectric charge. A key insight was recognizing that a distinctive feature of otherwise dissimilar materials is that they can attain and maintain either positive (nylon, glass, silicon, acrylic, bakelite, mica, cotton) or negative charge (PTFE, PVC, vinyl, saran, polyethylene, polypropylene, polyester, PLA, polyurethane, ebonite)^81–90^. A characteristic of triboelectric phenomena is that many materials that are neutral in principle can acquire electrical charge by transferring them to another location, repeated movement, or friction. Static friction arises on solid surfaces from features of surface roughness across multiple length scales, and the resulting acquired electrical charge can survive for a fairly long times^91,92^. Comparison of adhesive capabilities of Sgs-glue using this lens revealed that the positive electric charge of the material or surface is the primary factor responsible for successful adhesion. Surfaces with positive electric charge are better adhesive materials, whereas surfaces possessing negative triboelectric charge are poor adhesive materials. Surface features able to acquire charge, known as asperities, are present down to nano-scale dimensions, and, in the context of Sgs-glue adhesion to a positively charged surface, would result in true solid-to-solid contact existing only at a limited number of points. This accounts for the glue plaque adhering to only a fraction of the nominal area of contact. Additional contributions to understanding of the bioadhesion process can be gleaned from studying the distribution and properties of local asperities on different surfaces at different slopes.

Multiple independent observations suggest that larvae posess a signaling system able to evaluate the electrical charge associated with a potential substrate for gluing and/or mediate electrostatic interactions associated with gluing. First, it is commonly observed by drosophilists that cultures of wild-type strains of *D*. *melanogaster* and other species (*e*.*g*., *D*. *mauritiana*, *D*. *ananassae*, and *D*. *rajasekari*) as well as numerous mutant strains often prefer a cotton plug for pupariation over a material with a large and uniform smooth surface^42^. Cotton and other fabrics (Table 1), including the cotton wool used by many drosophilists, can acquire a long-term positive triboelectric charge during fabrication and processing. Second, we observed that prepupariating larvae „quietly sitting “ on an ebonite (sulfur-vulcanized natural rubber) surface „jump” away if the opposite-side surface is rubbed with fox fur. In this special case of the friction effect on triboelectric charge, ebonite temporarily acquires one of the strongest known aquired negative electrostatic potentials (-2 to -4 V)^81,82,93^.

**Table 1:** List of web links with assembled triboelectric series based on various independently tested materials.

https://www.alphalabinc.com/triboelectric-series/
https://www.htae.net/checklist/triboelectric-series-or-effect/597/
https://studylib.net/doc/18333423/substances-in-electrostatic-triboelectric-series
https://rimstar.org/science_electronics_projects/triboelectric_effect_series.htm
https://spicerart.com/wp-content/uploads/2020/06/An-Introduction-to-the-Triboelectric-Series.pdf
https://www.carolina.com/teacher-resources/Interactive/the-triboelectric-series-an-introduction-for-static-electricity-labs/tr35107.tr, http://soft-natter.seas.harvard.edu/index.php/Triboelectric_series
https://eesemi.com/tribo_series.htm, https://fraser-antistatic.com/knowledge-centre/insights/triboelectric-series-table/, https://fraser-antistatic.com/wp-content/uploads/2020/10/Triboelectric-Series-Iss.1-EN.pdf
https://www.sciencelearn.org.nz/images/3879-triboelectric-series, https://instructional-resources.physics.uiowa.edu/demos/5a1015-triboelectric-series
https://staticworx.com/esd_glossary/triboelectric-series/
https://www.rfcafe.com/references/electrical/triboelectric-series.htm
http://physicsed.buffalostate.edu/SeatExpts/resource/tribelec.htm
https://www.researchgate.net/figure/Triboelectric-series-short-summary_tbl2_326959935

While positive charge is not the only important feature, these two examples suggest that it is assessed by the wandering larvae in selecting a pupariation site. In nature, *Drosophila* prefer to pupariate close to a food source and in a conspecific cluster of pupariating animals^43^. A signaling system to assess positive charge would provide a mechanism for the formation of conspecific clusters. The ability of *Drosophila* to adhere intensely to chitin of phylogenetically very distant insect species (*Tenebrio* and *Galleria*) indicates that, under laboratory conditions, *Drosophila* can also aggregate in heterospecific clusters. Interestingly, the accessory glands of *Opodiphthera* moths females produce a secretory fluid that sticks their eggs to each other and then also to the substratum^94^. This suggests that the sticky-self-adhesion strategy observed for Sgs-glue and chitin may be a more widespread evolutionary strategy than previously appreciated, and that individual examples can evolve independently.

Our measurements identified three distinctive features that play an important secondary (to triboelectric charge) role in adherance: surface topography, pH, and wettability. Glue plaque showed some adherence to extremely smooth materials that are slightly negatively charged due of surface polishing, such as phosphor-doped silicon wafers. Purely smooth uncharged surfaces of PTFE, PVC, vinyl, polyethylene, polypropylene, ebonite etc. showed poor adherance, however, and a mild positive charge on smooth acrylic surface was insufficient for strong adherance. It may be that the local charge distribution or the chemical composition affects glue adherence for materials with mild triboelectric charge. We consistently observed greater adhesion to the rougher (bottom) side of silicon wafers (see Fig. 5e). In marine mussels, rough or chemically heterogeneous surfaces are also preferred^95,96^. Therefore, we suggest that roughness in triboelectrically favorable materials can increase the area of contact surface with the glue, thereby increasing its adhesion.

When we measured the pH of solid materials using previously published protocols^97–99^, the most favorable materials were alkaline or near neutral, whereas unfavorable materials were acidic (*e*.*g*., ebonite, most woods) (Fig. 5l). Since the pH of expectorated Sgs-glue is also alkaline (pH 8.7 to 9.1), neutralization via an acidic adhesion substrate would inhibit adhesive and polymerization reactions necessary for curing. Hence, it is important that the net charge of most Sgs-glue proteins is negative so they bind to the positively-charged matrix. The importance of wettability was revealed when we measured the contact angle (CA) in test liquid droplets (see Supplement), which indicated that interfacial strength is affected by the similarity in the surface energy of the Sgs-glue and its substrate. Materials with strong covalent chemical bonds, such as metals and some minerals, have surface energies that are too high to be determined by CA measurements. These materials showed poor adhesion to Sgs-glue, especially compared to materials with lower surface energy (Fig. 5f-5j and 5l). Since wetting is important when solids are brought into contact with a liquid and for the movement of micro-droplets on solids, a liquid Sgs-glue (*i*.*e*., pre-solidification) must wet a substrate’s surface to rapidly interact with it. Important to electrowetting of surfaces are a dielectric moment, dispersion of adhesive, and acid–base interactions^100^. The dielectric moment and acid–base interactions are mostly affected by pH. Two examples are the adhesion of mucin to polyether-modified polyacrylic acid, which is optimal at pH 4 to 5^101^, and dependence on pH of the rate of curing and bioadhesive properties of dopamine functionalized polyethylene glycol hydrogels^102^. A third example is the adhesion of mussel foot proteins to substrates, which occurs underwater. Each foot protein may require a different pH, so maintaining optimal redox stability in an oxidizing environment is crucial for adhesive function^103–106^. The type and concentration of electrolytes and pH are important contributing factors to the interfacial behaviour shown by these proteins during the adhesion/adsorption process^107^. Surfactants are usually required to facilitate a dielectric moment, and the dispersion of adhesive and/or acid–base interactions^100^. Expectoration of the Sgs-glue by the pupariating larva is fast, and solidification of this viscoelastic solute is even faster. To achieve such a rapid and complex set of reactions, the larva most probably provides the glue with a proteinaceous or other surfactant. It will be important to identify which of the eight currently known Sgs-proteins^6^ serves this function or whether it is provided by a smaller organic molecule(s). It is apparent from the studies of Voigt and Gorb^108^ and Lutz et al.^109^ that the proteinaceous content of the secretion helps wetting on difficult surfaces by decreasing interfacial energy and reducing the viscosity of the phases to enhance delivery of the adhesive.

Finding an extremely well-preserved GFP signal within solidified glue indicates that glue curing leads to the strong structural stability of (at least) the Sgs3 protein. It also suggests that polymerization of the glue occurs by mutual interactions or covalent reactions of individual Sgs-proteins. Although further chemical analysis of the Sgs-glue will be presented in a separate manuscript, numerous proteins present in the Sgs-glue are rich in cysteine residues, many of which are predicted to form disulfide bonds^6^. Different degrees of disulfide bond formation, or sets of preferred disulfide bond formation between selected Sgs-proteins may explain the both why plaque is homogenously smooth and the formation of the fingerprint. In addition, although the volume of expectorated salivary glue secretion is small (40 to 70 nl per gland), its extremely fast solidification within a few seconds could be explained also by a polymerization process. The so far identified Sgs-proteins are unique in nature, and lack homologs in the animal kingdom, so we know very little about their function. Therefore, one possibility is that one or more of the Sgs-proteins also possesses enzymatic activity, which upon contact with atmospheric oxygen and an initiator/ligand (*e*.*g*., exoskeletal chitin), drives the process of instantaneous glue polymerization.

Considering the data from multiple perspectives provides insights into the complex interplay between factors influencing adhesion. In addition to triboelectric charge, the wettability, topography, and acido-basic properties of a substrate are important considerations for Sgs-glue adhesion. These four recognition criteria have mutually dependent relationships. Consider wettability as an example. It is not a major or dominant criterion, because a surface that is not so easy and instantly wet (*e*.*g*., the polished side of the silicon wafer) can still be a very good adhesive substrate due to its positive electric charge-reproducible distribution of positive charge at the atomic level may facilitate strong electrostatic interactions with the glue. In contrast, different types of wood that are easy to wet are poor adhesive surfaces. This suggests that other factors, such as the long-term, if not permanent, presence of sap components including tannins, catechins, lignins, and polyphenols that have plant defense activities can also act as repellents^110–113^. Observations on prepupariating-larval behavior indicate that these animals can assess and recognize the suitability of several factors important for favorable pupariation/adhesion, and try to avoid unsuitable surfaces where Sgs-glue adhesion is low. This is an excellent example of premetamorphic or prepupariation deterrence, which has not yet been studied. Deterrence in insects is usually associated with preingestion detection of adverse factors, mainly in herbivorous species^114–118^. It will be important to understand how prepuriating *Drosophila* larvae sense electric charge, chemical composition, and surface topography of potential substrates and then use that information to choose a site for pupariation.

Observations on a few exceptional materials have broadened our understanding of bioadhesion and suggested that further research will reveal additional factors that define favorable conditions for glue adhesion. First, maple leaves were the most adhesive among the leaves we tested. Observations of Jie et al.^119^, Feng et al.^120^ and Ding et al.^121^ suggest that this results from their ability to act as triboelectric nanogenerators when they harvest mechanical energy from low-frequency motion to generate long- or short-lived static surface charge. This generation of positive triboelectric charge^122,123^ appears to be species-specific. Second, agates and marble are exceptional among stones. Blue agate shows modestly favorable adhesion, whereas dark-red and green agates are unfavorable (see Fig. 5j). Blue agate often contains potassium ferricyanide and ferrous sulphate. Atoms of iron can gain positive charges, and an interaction with atmospheric oxygen can increase the ratio of ferrous ions from iron oxide (FeO), stabilizing positive charge on agate surfaces^124^. In contrast, marble (metamorphic rock composed of recrystallized calcite and dolomite) is a very unfavorable substrate for adhesion (see Fig. 5j). This can be explained by the negative charge it obtains from calcium ions (adsorbed from the air) onto its surface^125^.

Future work should address two important aspects of glue adhesion. First, it will be important to understand if Sgs-glue released onto surfaces other than soda-lime glass produces diversiform structures. Such experiments will be useful to understand the relationship between material surfaces and plaque morphology and topography. Second, it will be important to quantify how the size of the attachment area of Sgs-glue influences adhesiveness. Under our experimental conditions, animals could attach freely to a relatively large and uniform surface area (usually 75 × 25 mm), and animals attached to corners were excluded to standardize conditions. In nature, however, larvae often pupariate in much smaller areas, and on the surface of materials that are not homogenous or uniform in composition (component distribution, local presence of dirt and/or impurities, etc.) and topography (smooth or rough). Moreover, experimental measurements in conditions standardized for temperature and humidity can be quite different from natural condition. Therefore, it is important to consider that adherence in nature may be affected by these and other factors, and that our adhesion measurements cannot be freely extrapolated to similar materials in nature.

Our findings show differences from previous work. Borne et al.^35^ found that the median contact area between the glue and the substrate was 1.10 mm^2^, leading to an adhesive strength ranging from 137 to 244 kPa. Our median value was two-fold greater: 2.5 mm^2^. While this dissimilarity may be due to the different methods used to perform area measurements, our adhesive strength data for soda-lime glass (189.76 kPa) lies within the previously reported range. This suggests that the observed differences in area may reflect methodological approaches. Furthermore, our observation that individual larvae perform slightly different individual peristaltic and head movements during pupariation, and for different times that can range from 5 to 20 s, identify a mechanism underlying variability in such measurements, as these behaviors will result in different sizes of Sgs-glue plaque and fingerprint area. Nonetheless, using standardized experimental conditions has allowed us to make internally consistent comparisons.

### Assessing the distribution of Sgs-glue on puparial cuticle uncovered a novel anatomical structure: bidentia

We showed that Sgs-glue spreads on the surface of the lateral segments in Y-shaped structures, which we name here bidentia. In fully formed puparia, these structures do not have cavities and are not capillaries (Fig. 6m-o and q). Bidentia documented using light microscopy and SEM were readily apparent on the puparial case, and visible in late-third instar larvae. In the larva, bidentia are nested inside elliptic or mandorla-like tubercles (small fleshy processes) resembling epaulettes when viewed under SEM. These tubercles, previously described as lateral fusiform areas or lateral creeping welts^126,127^, are present on the lateral side of each abdominal segment of all larval instars in multiple *Cyclorrhapha* species. Abdominal peristaltic movements during pupariation, followed by puparial case hardening and melanization, may increase the visibility of bidentia in puparia. However, we were unable to identify any similar structures in second-instar or younger larvae (see Fig. 7d,e,g,h,m,n,o).

This indicates that the formation or manifestation of bidentia is strictly associated with the transition from second to third (last) larval instar, and reflects the developmental commitment of the animal to undergo metamorphosis. At the same time, during pupariation, when bidentia become manifested, the tubercles become flaccid and morphologically repressed. Moreover, along with mandibular hooks, anterior and posterior spiracles, tracheal trunks, caudal sense organs and dorsal spinule raws that all continuously develop from the embryonic period (and provide typical characters for each instar), bidentia can serve as unique and distinctive morphogenetic features of the last larval stage. They appear to be formed and harden at the same time as the operculum is formed. In this respect, they resemble integumental scales (cuticular protuberances) that are observed in different insect orders and serve as pivotal markers of the premetamorphic larval stages, and whose appearance is associated with the culmination of allometric growth^128–134^.

Small interruptions or cracks in the Sgs plaque indicated that the glue is more rigid and fragile, whereas the chitinous cuticle is more flexible or elastic, after exposure to mechanical tension provided from pressure on the glass coverslip. Indeed, these are generally accepted properties of chitin in the role of arthropod’s exoskeleton^135–137^. Because the cuticle below these cracks in the glue remained uninterrupted, it provided good evidence that the Sgs-glue spread onto the surface of the bidentia. Thus, we conclude that the viscoelastic glue creeps up the surface of the lateral cuticle. This phenomenon may reflect phoretic liquid dispensing or microfluidics on chitinous cuticle, which can be due to a balance between adhesive and cohesive forces. The adhesion enables watery glue to „climb “ upwards against gravity, whereas cohesive forces are responsible for surface tension, a phenomenon that results in the tendency of a liquid’s surface to resist rupture during motion. Because the surface of the cuticle, even in a pupariating larva, is not that of a typical capillary tube, the upward motion of the glue cannot primarily be due to capillary forces, but reflects the perfect balance between the adhesive and cohesive forces of the liquid before it solidifies. Strong cohesive forces, due to their ability to form hydrogen bonds with one another inside a water-based solute, tend to minimize adhesion. However, the proteinaceous components of the aqueous glue also affect cohesive forces within the liquid and modify the adhesive forces towards the cuticle to generate this balance.

We highlight three plausible, not mutually exclusive, explanations for why Sgs-glue moves exclusively via the bidentia cuticular structures. (1) Insect cuticle, including that of *Drosophila*, contains various waxes secreted onto its surface^138–146^. Water does not wet waxed surfaces because the cohesive forces within the water are stronger than the adhesive forces between the water and the wax. Water can wet, to a limited extent, a polysaccharide and/or proteinaceous surface, and spread out on it because the adhesive forces between the liquid and that surface can be measurably stronger than the cohesive forces within the water. If bidentia surfaces contain less wax, liquid glue would spread further through them. (2) Bidentia are clearly distinctive structures. They may contain structural component(s)—unique protein(s) that specifically interact with protein component(s) of the glue to provide chemotactic spatial guidance to Sgs flow. (3) SEM observations of larvae prior to pupariation show that the bidentia are partially invaginated. This could facilitate the flow of the liquid glue via local impressions. Their role requires further study, as it was technically impossible to obtain samples of pupariating and peristalsis-performing animals to see whether the localized invaginations seen in larvae were still present or had already changed to a concave form.

It is unclear why, in most puparia, the GFP-Sgs signal in the posterior bidentia branches is longer than in the anterior branches, as the sibling branches of bidentia appear to be equilateral. When the length of these branches is measured using the GFP signal, it is shorter than when measured using light microscopy or SEM. This could be explained if the Sgs does not spread over the entire length of the bidentia and its spread depends on the amount of glue expectorated and the speed of its solidification. In turn, local variation in the amount of the glue that is expectorated can be explained by variability in the contractile peristaltic waves. This would cause the Sgs-glue to reach the anterior and posterior bidentia branches at slightly different times. This could be due to the amount of glue released by the pupariating larva and how thoroughly the ventral side of the cuticle is covered while the liquid is still glue. Even local humidity could affect the outcome, as under slightly drier conditions, viscoelastic glue will start solidification earlier and prevent further expansion of the glue into the bidentia.

It is somewhat surprising that bidentia and other structures on lateral segments were neglected for over 100 years of *Drosophila* research, and to our knowledge have not been mentioned or described before. They are most probably differentiated during embryonic development like other cuticular structures. The work of Kuhn et al.^147^ indicates that certain cuticular characteristics of *D*. *melanogaster*, such as ventral denticles and dorsal spinules, change their morphology with each larval molt. While trunks of the bidentia stem from the sternital region (ventral side) of the segment among denticle belts, they have not been described in any seminal papers or reviews devoted to this or related topics^5,35,148–151^.

From a genetic perspective, bidentia, like other ventral segment patterns such as denticle belts, must be determined by specific set of gene pathways, and must differentiate during embryogenesis. We propose that the function of bidentia are achieved by a bidentium-specific component(s) that enables highly efficient binding of the glue and fortifies the stability of the puparium to the substrate. Thus, when Sgs-glue spreads, it is able to embrace the exoskeleton and increase its stiffness and resistance against mechanical force during pupal/adult development. Functionally, bidentia contribute to preventing the removal of the puparial case from the substrate. The bands of the Sgs-glue partially surrounding the puparial case (seen as GFP signal) act much like carton-strapping metallo-plastic tying belts able to hold a „package “ during transport. Interestingly, larvae of the New World saprophagous fly, *Nausigaster unimaculata*, and perhaps other species of this genus (*Syrphidae*), smear themselves with a fluid emitted from their anus, and blow bubbles into it with their head pump. It takes only a few seconds for this material to dry into a hard, white substance that coats the entire puparium, except for the spiracles^152^. The functional significance of this hard coating is unclear, but handling of *Nausigaster* puparia reveals a high level of mechanical robustness, and thus the author speculates that these kinds of coatings supply some level of protection to the forming puparium. Moreover, pupation of the army cutworm caterpillars (*Euxoa auxiliars*) occurs in the soil, in a cell prepared by the larva. The walls of the cell are formed with salivary secretion, which hardens and provides a necessary degree of rigidity and protection^153–155^. Thus, this mechanism of salivary or anal gland-elaborated hardening secretory cement appears to be repeatedly and independently used in the evolution by unrelated species and even in phylogenetically distant orders of insects. The known *Sgs* genes of *Drosophila* are unique in their sequence and amino acid composition of encoded proteins and do not have orthologues in other insect groups^6,156^. Therefore, this kind of „interaction “ between two types of excretory composite matrices (chitinous cuticle and Sgs-glue) would seem to require close coevolution between genes encoding Sgs-glue and bidentium protein components in the *Drosophila* genus, and warrant further investigation. Recently, we described^27^ species of *Drosophila* that, in contrast to *D*. *melanogaster,* not only do not pupariate on the wall of culture vials (some pupariate in the food), but some even that do not produce and/or secrete any Sgs-glue. From a gene-evolution perspective, it would be insightful to investigate whether bidentia or similar structures are also formed in species that may have fewer or no *Sgs*-genes and that show strikingly different pupariation strategies.

### Concluding remarks

Studying the gross morphology and mechanical properties of released larval *Drosophila* Sgs revealed that this rapidly solidifying cement forms a distinct enamel-like plaque composed of a central fingerprint and laterally layered cascading terraces. Solidifying glue, which has an alkaline pH (8.7 to 9.1), rapidly produces crystals of KCl on these alluvial-like cascading terraces. Testing more than 80 different substrates for adhesion revealed that Sgs-glue favors positively charged and wettable (hydrophilic) surfaces for strong adhesion and rejects negatively charged and hydrophobic surfaces with an acidic pH. The primary recognition factor for successful adhesion of the Sgs-glue is a positive triboelectric charge on the material surface.An interesting feature of the expectorated glue is its ability to move upwards against gravity on the surface of freshly formed puparia, via specific and novel anatomical structures of the lateral abdominal segments that we named „bidentia”.

Positive triboelectric charge is the most important of the four factors that contribute to successful Sgs-glue adhesion and larval recognition of a suitable adhesion site. However, surface topography, wettability, and the material’s pH compete as important secondary recognition factors. These factors function contextually: if any has an overwhelmingly strong influence, it becomes the most important secondary factor. The triboelectric charge, wettability, pH, and mild roughness of a substrate ‘s surface, as well as the hardness and elasticity of the cured Sgs-glue plaque contribute the quality of bioadhesion. Understanding their roles provides bioinspiration for the development of novel adhesives.

Our recent in-depth proteomic analysis of salivary gland Sgs-glue secretion revealed it to have unexpected complexity, prompting us to question its significance. The current analysis comprises the first step in this quest. Here, we report baseline data on glue stiffness, strength, viscoelasticity, and fracture resistence, and insights into the fundamental properties of substrates required for good adhesion. We obtained all of these insights using a standardized mechanical tension device that we will be able to use to quantify changes in the properties of Sgs-glue and larval behavior after individual proteins and genes are modified using the molecular-genetic tools available in *Drosophila*. These experiments will allow us to dissect the biological basis of bioadhesives in a reproducible experimental system. Not only will we be able to dissect the contribution of individual *Sgs* genes, we will be able to perform phylogenetic comparative studies across different Dipteran species.

## Materials and methods

Can be found within online version of Supplementary Information including Supplementary Data.

### Supplementary Information

Supplementary Information.

## Data Availability

The data described in this manuscript is available within the manuscript and its Supplementary Information. Raw data is available by upon request from RF and does not require an MTA for access.

## Abbreviations

ABS: Acrylonitrile butadiene styrene
AFM: Atomic force microscopy
DIO: Diiodomethane
DTT: Dithiothreitol
EBD: Electron backscattered diffraction
EDS: SEM-coupled energy dispersive spectroscopy
EG: Ethylene glycol
GFP: Green fluorescent protein
KCl: Potassium chloride
LWAB: Lifshitz-van der Waals acid–base theory
*N*EMI: *N*-Ethylmaleimide
PLA: Polylactic acid
PTFE: Polytetrafluoroethylene (teflon)
PVC: Polyvinylchloride
SEM: Scanning electron microscopy
SG: Salivary gland
Sgs: Salivary glue secretion
TEM: Transmission electron microscopy
TOF-SIMS: Time-of-flight secondary ions mass spectrometry
