# Supplementary material for: Gross morphology and adhesion-associated physical properties of Drosophila larval salivary gland glue secretion
Source: Sci Rep. 2024 Apr 29;14:9779. doi: 10.1038/s41598-024-57292-8 (PMC11059401; doi:10.1038/s41598-024-57292-8)
Supplement: Supplementary file 1 — Supplementary Information. [file 41598_2024_57292_MOESM1_ESM.pdf]

GROSS MORPHOLOGY AND ADHESION-ASSOCIATED PHYSICAL PROPERTIES OF *DROSOPHILA* LARVAL SALIVARY GLAND GLUE SECRETION

Milan Beňo[1], Denisa Beňová-Liszeková[1], Ivan Kostič[2], Michal Šerý[3], Lucia Mentelová[4], Michal Procházka[5], Ján Šoltýs[6], Ludmila Trusinová[1], Mário Ritomský[2], Lubomír Orovčík[7], Monika Jerigová[8], Dušan Velič[8✝], Peter Machata[5], Mária Omastová[5], Bruce A. Chase[9, 10] and Robert Farkaš[1*]

[1]Laboratory of Developmental Genetics, Institute of Experimental Endocrinology, Biomedical Research Center v.v.i., Slovak Academy of Sciences, Dúbravská cesta 9, 84505 Bratislava, Slovakia, [2]Department of Sensor Information Systems and Technologies, Institute of Informatics v.v.i., Slovak Academy of Sciences, Dúbravská cesta 9, 845 07 Bratislava, Slovakia, [3]Department of Applied Physics and Technology, Faculty of Education, University of South Bohemia, Jeronýmova 10, 37115 České Budějovice, Czech Republic, [4]Department of Genetics, Comenius University, Mlynská dolina, B-1, 84215 Bratislava, Slovakia, [5]Department of Composite Materials, Polymer Institute v.v.i., Slovak Academy of Sciences, Dúbravská cesta 9, 84541 Bratislava, Slovakia, [6]Department of Physics and Technology at Nanoscale, Institute of Electrical Engineering v.v.i., Slovak Academy of Sciences, Dúbravská cesta 9, 84104 Bratislava, Slovakia, [7]Division of Microstructure of Surfaces and Interfaces, Institute of Materials and Machine Mechanics v.v.i., Slovak Academy of Sciences, Dúbravská cesta 9, 84513 Bratislava, Slovakia, [8]Laboratory of Secondary Ion Mass-Spectrometry, International Laser Centre, Slovak Centre of Scientific and Technical Information, Ilkovičova 3, 84104 Bratislava, Slovakia, and [9]Department of Biology, University of Nebraska, 6001 Dodge Street, Omaha, NE 68182-0040, USA, [10]Department of Data Analytics, Endeavor Health, NorthShore University Health System, Skokie, IL 60077, USA

Running title: Physical properties of *Drosophila* Sgs-glue

*Corresponding address: Robert Farkaš
Laboratory of Developmental Genetics
Institute of Experimental Endocrinology
Biomedical Research Center v.v.i.
Slovak Academy of Sciences
Dúbravská cesta 9
845 05 Bratislava
Slovakia
phone: (+421 2) 3229-5235
fax: (+421 2) 5477-4284
E-mail: ueenfark@savba.sk

SUPPLEMENTAL DATA

Contact angle measurements

To understand the role of wettability in the adhesion of Sgs-glue, contact angle (CA) measurments were performed on selected substrates. With measurements of the contact angles of multiple test liquids of different polarity, the surface energy of solid materials can be calculated, including the dispersive and polar or acid-base components. The compatibility of surface energy between two substrates is expected to play a significant role in the interfacial strength of materials such as composites, when physical interactions dominate over chemical bonding[157, 158]. It is important to note that not all substrates are appropriate for CA measurements. Those with high surface energy *e.g.* metals and minerals in which CA shows complete wetting when the surface is sufficiently clean[159, 160]. Therefore, from all the tested materials, for CA measurements and CA hysteresis calculations, only ten materials were selected as low-energy surfaces. For these low-energy surfaces, the surface energy (γ) is usually in the range from 10 to 50 mJ/m^2 due to the dominance of bonds with low-binding energy such as dispersive, van der Waals or hydrogen bonds. Materials, where strong covalent chemical bonds dominate, exhibit high surface energy (500 to 5000 mJ/m^2)[159, 160].

The series of contact angles of ten substrates is shown in the Figure S4. It should be noted, that a single CA value corresponding to the Young CA can be achieved only theoretically for an ideal solid surface. For real samples, CA always varies between the two limiting values - advancing (highest) and receding (lowest) CA, due to CA hysteresis. Since a single CA value can often provide misleading information about wettability[161], the values of both, advancing and receding CA are listed in the text insets of all drop pictures in Figure S4. Moreover, the advancing Θ_A and receding Θ_R contact angle measurement can be used to estimate the most stable CA Θ_{MS} necessary for the surface energy calculation using equation (1)[162] as originally proposed by Andrieu *et al.* [163]:

$$cos\theta_{\mathrm{MS}} = \frac{cos\theta_{\mathrm{A}} + cos\theta_{\mathrm{R}}}{2} \quad (1)$$

This approach provides reproducible data, and also more accurate results than the frequently used static CA approach[161, 162]. We followed the instructions written in a protocol for reproducible measurement of the advancing and receding contact angle, published by Huhtamäki *et al.*[161]. The surface energy was calculated using CA hysteresis values of three liquids with different polarities - water, ethylene glycol (EG), and diiodomethane (DIO). The Lifshitz-van der Waals acid-base theory (LWAB) was used for surface energy calculation using the surface energy components proposed by Good and Van Oss[164] (Table S2) and Schrader and Loeb[165]. This method provides the most comprehensive information about the surface free energy of solid surface. The calculated surface energy values are listed in Table S3.

Figure S5 documents the comparison of surface energy components calculated using the LWAB method and Sgs-glue adhesion for ten different substrates. The results show a positive correlation between Sgs-glue adhesion and absolute values of surface energy with the exception of viton, and polystyrene. This indicates that the surface energy similarity between Sgs-glue and its substrate plays a role in the interfacial strength, but that it is not the only important factor. Materials with strong covalent chemical bonds, such as metals or minerals, exhibit too high of a surface energy, which cannot be determined by CA measurements. Nevertheles, this group of materials performed as poor adhesion substrates for Sgs-glue, when compared to the majority of low energy materials which were analyzed by CA measurements. These results indicate, that in addition to unfavourable triboelectric properties, the surface energy of metals or minerals is too high to provide good conditions for adhesion of Sgs-glue, suggesting that surface energy compatibility is an influential secondary factor influencing adhesion strength.

In addition to a positive triboelectric charge, the value of the surface energy can help to explain the higher adhesion of Sgs-glue to mica muscovite compared to other minerals. Unlike other minerals classified as high energy surfaces, the adjacent layers of structural unit in mica muscovite

are weakly bonded by van der Waals interactions[166], lowering their surface energy to a measurable value.

The highest surface energy value from the tested materials was obtained for unmodified silicon wafers, which was higher than the value calculated for B-doped silicon wafers (see Table S1). Taking into account only surface energy values, this could mean that the surface energy of unmodified silicon wafers differs more from the surface free energy of Sgs-glue and therefore is less compatible than B-doped silicon wafers. However, this aspect needs to be considered as a secondary factor to triboelectric charge, which is the most significant factor for adhesion in silicon wafers.

It should be noted that it is challenging to directly compare contact angle and surface free energy values with those published previously in the literature. This is most probably the results of random effects leading to a lack of reproducibility in measurement of the static contact angle approach[167] as well as other factors such surface roughness or contamination, which will affect wettability[159, 160].

SUPPLEMENTAL TABLES

Table **S1**. Advancing, receding, and most stable contact angles of water, diiodomethane (DIO) and ethylene glycol (EG) on selected substrates.

		Θ_{A} (°)			Θ_{R} (°)			Θ_{MS} (°)
**PTFE**	Water	116.1	±	1.2	96.7	±	3.5	106.2
	DIO	90.4	±	2.7	60.2	±	1.8	75.8
	EG	92.1	±	2.1	75.6	±	1.3	83.9
**poly lactic acid**	Water	95.3	±	1.3	45.0	±	2.3	72.1
	DIO	110.5	±	2.6	39.5	±	1.4	77.8
	EG	65.1	±	1.7	16.7	±	0.7	46.4
**polypropylene**	Water	97.1	±	1.6	75.7	±	1.2	86.5
	DIO	59.3	±	3.5	48.8	±	0.4	54.2
	EG	71.6	±	0.7	40.9	±	3.5	57.6
**viton**	Water	99.6	±	1.7	50.9	±	0.9	76.6
	DIO	74.9	±	1.1	29.9	±	1.4	55.7
	EG	75.5	±	0.8	28.4	±	2.0	55.6
**bakelite**	Water	98.7	±	0.7	35.5	±	0.4	70.6
	DIO	56.7	±	2.1	32.4	±	1.1	45.8
	EG	73.5	±	0.4	16.5	±	0.6	51.6
**polystyrene**	Water	90.3	±	1.8	50.5	±	0.5	71.6
	DIO	80.9	±	1.4	31.9	±	0.7	59.8
	EG	47.2	±	0.9	20.2	±	1.8	36.0
**mica muscovite**	Water	44.5	±	1.0	10.2	±	0.4	31.9
	DIO	44.5	±	2.1	20.7	±	2.5	34.5
	EG	38.3	±	2.0	9.1	±	0.7	27.6
**kapton**	Water	96.8	±	0.8	55.4	±	3.2	77.0
	DIO	53.7	±	1.6	10.1	±	1.4	38.0
	EG	59.3	±	0.7	13.6	±	2.1	42.2
**B-doped silicon**	Water	47.2	±	2.1	15.5	±	1.8	34.8
	DIO	45.5	±	0.4	33.5	±	0.9	39.9
	EG	32.1	±	3.1	10.5	±	0.7	23.8
**undoped silicon**	Water	38.1	±	1.4	20.2	±	2.1	30.4
	DIO	46.0	±	0.5	22.1	±	1.8	35.8
	EG	10.0	±	0.8	0.0	±	1.4	7.1

Table **S2**. Surface energy values of the test liquids and their components, obtained according to Good and Van Oss[164].

**Test liquid**	**γ (mJ/m^2)**	**γ^{LW} (mJ/m^2)**	**γ^{AB} (mJ/m^2)**	**γ^+ (mJ/m^2)**	**γ^- (mJ/m^2)**
water	72.8	21.8	51.0	25.5	25.5
ethylene glycol	48.0	29.0	19.0	1.92	47.0
diiodomethane	50.8	50.8	0.0	0.00	0.0

Table **S3**. Surface energy components of samples calculated according to the LWAB model; ordered from lowest to highest value.

**Substrate**	**γ (mJ/m^2)**	**γ^{LW} (mJ/m^2)**	**γ^{AB} (mJ/m^2)**	**γ^+ (mJ/m^2)**	**γ^- (mJ/m^2)**
**PTFE**	26.92	26.92	0	0	0.09
**poly lactic acid**	31.53	18.16	13.37	3.34	13.36
**viton**	33.92	31.05	2.87	0.19	10.82
**polypropylene**	34	31.9	2.1	0.32	3.47
**bakelite**	37.68	36.58	1.1	0.02	14.96
**polystyrene**	37.83	28.69	9.14	2.3	9.09
**mica muscovite**	42.59	42.29	0.3	0	53.22
**kapton**	43.48	40.6	2.88	0.38	5.37
**B-doped silicon**	44.55	39.66	4.89	0.12	48.34
**undoped silicon**	48.59	41.66	6.93	0.25	48.34

MATERIALS AND METHODS

Fly culture and staging

Wild type (*Oregon R*) and *Sgs3-GFP* fruit flies (*Drosophila melanogaster*, Meigen) were cultured in 50 ml vials or 200 ml bottles at 23°C either on agar-yeast-cornmeal-molasses medium[168, 169] or commercial Bloomington-Formula Nutri-Fly food (Genesee Scientific) with the addition of methylparaben to prevent molds. The *Sgs3-GFP* construct is a fusion of the jellyfish GFP coding sequence behind the 1.8 kb of the *Sgs3* gene carrying its upstream regulatory information and the first third of its protein coding sequence truncated after nine tandem repeats[34]. These sequences were fused in-frame into a *pCaSpeR-4* vector[170] and used to generate transformed flies[171]. The *Sgs3-GFP* stock we used has insertion in 3rd chromosome, and was obtained from Andy J. Andres (University of Nevada, Las Vegas, NV, USA).

For a small subset of experiments, the chitinous exoskeleton of two insect species, *Tenebrio molitor,* and *Galleria mellonella*, was used. Yellow mealworm beetle (*Tenebrio molitor*, Coleoptera: Tenebrionidae) was reared on wholemeal flour mixed with wheat bran and middlings at 27°C and 60% RH supplemented with drinking water as described by Stellwaag-Kittler[172] and Williams and Caveney[173] or as described previously[174]. The greater wax moth (*Galleria mellonella* L., Lepidoptera: Pyralidae) was reared on honey-honeycomb wax-bran-Gerber cereal-glycerol-yeast mixture at 30°C and 50% RH according to Sehnal[175]. The elytron of recently deceased adult *Tenebrio molitor* and pupal exuvial cuticles of *Galleria mellonella* were used as heterologous sources of chitin in pull-off force experiments.

Cleaning microscope glass slides and cover slips

Because various manufacturers provide different qualities of glass slides and cover slips, even when the slides are pre-cleaned, we cleaned all slides by overnight immersion in chromic-sulfuric acid, a very intense rinse in tap water, followed by 1 hr ultrasonication in 2% detergent (regular dish washing-grade), a second very intense rinse in tap water, a rinse in deionized Milli-Q water and a brief rinse in pure ethanol followed by air drying in a dust-free environment.

Morphological observations (examinations) of released and solidified glue

To perform very basic morphological observations of the released glue, wandering third instar larvae were allowed to find their optimal place for pupariation on microscope glass, and release their glue via expectoration. Within 15 to 20 seconds afterward, the freshly formed prepupa was quickly but gently removed from the glue. The solidified glue was then kept at room temperature (23°C) in a dust-free environment for at least 1 hr. When necessary, glue samples were stored in a Petri dish with a silica desiccator for hours or days before examination without any apparent change of their appearance. To avoid any unwanted side effects, samples were not processed, and the Sgs-glue was used for further observations such as it was.

To view the Sgs-glue under bright field, phase contrast, Nomarski interference, and polarized illumination, images were taken at various magnifications on a Leica DM6000B upright research

microscope equipped with a Leica DFC320 digital camera, operating with Leica LAS 3.1.0 software. Where necessary to improve the depth of field from combined several independent images, a Zerene Stacker *v.* 1.04 was used, and the contrast threshold was set to 30%.

The GFP signal from the *Sgs3-GFP* construct was examined by using a Leica MZ16F-A/X stereomicroscope with a GFP1 filter and images taken using a Leica DFC480 digital camera, operating with Leica LAS 3.6.1 software. For higher-magnification observations of *Sgs3-GFP* glue, the fluorescent mode of a Leica DM6000B microscope equipped with a Leica DFC320 digital camera was used.

To measure the area, on the acquired image while displayed in LAS Analysis Module, the region of interest (ROI) was drawn as an irregular shape around an object (Sgs-glue plaque) by clicking, holding, and dragging around the object to be measured. The area within was measured using an interactive measurements tool (see Figure 2a).

Sgs (glue) plaque processing for SEM

The Sgs-glue was obtained as described above and allowed to attach to pieces of glass cover slip. They were either kept (under dry conditions) until used for direct SEM observation without further processing (for environmental SEM mode) or processed as follows. In contrast to our previously used procedure[176], we found that fixation in 4% paraformaldehyde + 2% glutaraldehyde in 100 mM sodium cacodylate, followed by osmium tetroxide postfixation and dehydration in ascending ethanol series introduced unwanted side effects *e.g.*, dissolving previously unnoticed glue components. We also omitted critical point drying and found that the solidified glue can be directly and safely sputter-coated (Balzers SCD-030) with gold-palladium or platinum at 35-40 mA and a pressure of 0.05 to 0.1 mbar to produce a 40-50 nm-thick continuous alloy layer that prevents charging effect during scanning. Samples were imaged on a FEI Quanta FEG250 scanning electron microscope with the field-emission cathode at an accelerating voltage of 10 or 15 kV. Some samples were inspected in the environmental mode that eliminates charging by introducing water vapor into the SEM chamber. In such a mode, metal coating of samples is not needed. An accelerating voltage of 7 kV was used for operating in the environmental mode, and the usual working distance of 10 to 11 mm was extended to 17 to 20 mm. To measure any parameters, including crystal dimensions, the Measurement/Annotation tool of FEI xTm software v. 6.2.8.3161 was used.

Processing of puparial cuticles and whole larvae for SEM

Completely clean larvae of 1st, 2nd, or 3rd instar and puparial cases were fixed in 4% paraformaldehyde (Polysciences # 18814) + 2% glutaraldehyde (Ted Pella # 18426 or Serva # 23115) in 100 mM sodium cacodylate (Serva # 15540) (pH 7.2) for several days at room temperature. Samples were rinsed 6 × in 100 mM sodium cacodylate (pH 7.2), 20 to 30 min each at room temperature. To facilitate further processing and improve the flattening of the soft-bodied larval exoskeleton and minimize their distortion, some of them were dipped (20 sec to 5 min) into a super-skipper[177, 178] after fixation. Although about 15% of animals in our tests showed improved flattening, this protocol was not as successful and reliable as desired, and the majority of samples were processed without a super-skipper solution. Samples were then postfixed in 1% osmium tetroxide (EMS # 19134) in H_2O for at least 7 days, and extensively rinsed in H_2O (minimum 6 ×

for 20 min each). Samples were dehydrated in an ascending series of ethanol (Merck # 1.00986) (30%, 50%, 70%, 96%, and 100%) for 30 min each; the dehydration step with 100% ethanol was repeated twice before applying a mixture of 100% ethanol + 100% acetone (Merck # 1.00014) (1:1) twice, followed by three changes of absolute acetone. Addition of hexamethyldisilazane (HMDS; Sigma # H-4875) in place of Peldri II to facilitate critical point drying[176, 179], was done in several steps to keep samples permanently wet and avoid drying: the first volume of HMDS was applied in the presence of the small remnants of acetone; then, after 20 to 30 min, the HMDS was quickly removed and fresh HMDS added; finally, samples were kept in the HMDS for 30 min, and then remnants of the HDMS were allowed to evaporate completely.

Dried animals or cuticles were mounted on pieces of Scotch double-sided tape on 16 or 24 mm aluminum SEM stubs under a stereomicroscope. Samples were sputter coated for 2.5 min with gold-palladium using a Balzers sputter coater device SCD-030 at 35-40 mA per stub and at a pressure of 0.05 to 0.1 mbar to produce a 40-50 nm continuous alloy layer. Samples were viewed and photographed on a FEI Quanta FEG250 scanning electron microscope with the emission field cathode at an accelerating voltage of 10 kV. The obtained images were processed as described above. The bitmap images obtained were processed and labeled using Adobe Photoshop or Corel Draw software and assembled into figures using Adobe Photoshop. To keep the presentation of microscopic data uniform, the anterior end of animals is always oriented to the left, and the posterior end to the right, dorsal up and ventral down, regardless of the magnification.

Mechanical force measurements

Completely clean (extensively rinsing with tap and then distilled water, followed by brief drying) wandering 3rd instar larvae were allowed to search for their optimal pupariation site *ad libitum* on the microscope soda-lime glass slides (and the other tested material surfaces - see below) placed inside a large (Ø 20 cm) glass Petri dish. After they released Sgs-glue and pupariated, the glue was allowed to dry for another 1 hr, aged for 24 hr (at 23°C with 80% humidity), and then 2 mm zinc-chromium coated steel o-ring circles (Bossard BN 726 140HV; 7.5 mg each) were mounted on the dorsal side of the puparium using Loctite SuperBond (Henkel GmbH.) instant glue. Less than 0.1 μl of the Loctite SuperBond was applied on the dorsal side of the puparial case, the steel circle was placed onto it with demagnetized superfine Dupont tweezer # 4, and held 5-10 seconds with the tweezers until it hardened and the circle could stand independently (see Supplemental Figure 6a,b). Before any measurement (regardless of the age of the animal being studied), the Loctite SuperBond was allowed to harden further for at least 2 hr at room temperature. The same protocol was used for the adhesion of puparia to the other types of surfaces used as substrate materials listed below.

For mechanical adhesion force measurements (stiffness), a tensometric transducer (200 g capacity) with a HX711 processing module and 24bit AD converter connected via a L298N H-bridge (Geekcreit Dual H Bridge Driver Board) to a NAE016 stepper motor (bipolar 2-phase 4-wire DC 4-9V Drive Stepper Motor Screw with nut slider; 1,8 °/step eq. 0.025 mm/step) was used (see Supplemental Figure 6c,e). The resolution of the tensometer registered change was 0.01 g. The control and communication of the unit was mediated via an Arduino Nano modul connected to a PC via a USB port (Supplemental Figure 6d). The measured data were collected using our own software (ForceSketch) operating under 32-bit Windows XP or 64-bit Windows 7/10, and allowing force-time curves to be recorded in vector graphics with the data collected in ASCII format being fully convertible to Excel tables (Supplemental Figure 7). Subsequently, values of pull-off forces measured in grams were converted to Newtons (1 gram = 0.00981 Newton). Then, Newton values were used over the area in square millimeters (mm^2) to calculate the pressure using CalculatorEdge

(http://www.calculatoredge.com/new/pres.htm). Final values were expressed in kiloPascals (kPa). After the pulling experiment, the surface area of the glue from which the animal was removed (for all materials) was measured using the LAS Analysis Module within Leica LAS 3.6.1 software, as described above (see also Figure 2). We statistically evaluated the data from measurements of pull-off forces for a minimum of 15 single puparia in each experimental group, in triplicates (altogether over 5000 puparia).

Altogether we tested 83 different materials (85 different surfaces) for the adhesion to Sgs-glue. The following categories of materials were tested as gluing substrates for *Drosophila* larval Sgs secretion: [**A**] glass and related materials, [**B**] plastic polymers and related materials, [**C**] metals, [**D**] woods and paper, [**E**] plant leaves, [**F**] stones and minerals, [**G**] graphene, and [**H**] chitins.

More specifically these materials were tested:

[**A**] glass and related materials: soda-lime glass (microscope glass slides), silanized microscope glass slides, dithiothreitol (DTT)-treated microscope glass slides, silicons (P boron type of silicon 8-12 Ω/cm, non-boron P type silicon (Prolog Semicor Ltd., Kyiv, Ukraine), N-type silicon 10-12 Ohm.cm (Prolog Semicor Ltd., Kyiv, Ukraine), intrinsic/undoped neutral silicon >10000 Ohm.cm (Siegert Wafer GmbH., Aachen, Germany), HSQ (Hydrogen silsesquioxane) F_{Ox}25 silicon flowable oxide also known as $[(HSiO_3)_{2n}]$ (Dow Corning Inc., Midland, MI, USA), DNS P+CZ (B) type of silicon 0.005 to 0.015 Ω/cm (Dynamit Nobel Silicon Inc., Novara, Italy), spin-coated SU8-covered silicon (MicroChem Inc., Westborough, MA, USA).

[**B**] plastic polymers and related materials: clear solid polystyrene, polyethylene, polypropylene, acrylon (polymethylmethacrylate plexiglass), teflon (polytetrafluoroethylene, PTFE), polycarbonate, kapton, an electron beam resist polymethyl methacrylate (PMMA) $[CH_2{=}C(CH_3)COOCH_3]$ MW 450000 (Kayaku Advanced Materials/MicroChem Corp., Newton, MA, USA), acrylonitrile butadiene styrene (ABS), polylactic acid (PLA), polyoxybenzylmethylenglycolanhydride known as bakelite (Gummon, ETA Elektro-Praga Hlinsko, former Czechoslovakia), ebonite (Schönberger Ebonite Manufaktur GmbH., Hitzacker, Germany), viton known as high-performance fluorocarbon-based elastomer FKM6000 (Gumex Ltd., Žilina, Slovakia). All these plastic materials were in the form of sheets having the same size as the microscope slide (25 × 75 mm), and thicknesses between 1 and 3 mm.

[**C**] metals: aluminum, iron, stainless steel, zinc, gold, copper, brass, chromium, platinum, titanium, nickel, tin, lead, vanadium, tungsten, and silver. Except for chromium which was obtained from Nanofilms Corp. (Westlake Village, CA, USA), these materials were provided from the stocks of the Institute of Informatics of the Slovak Academy of Sciences, and a majority of them came from the local metal manufacturers or the own spin-off production of the Institute. All of these metals were also in the form of polished rectangle sheets the same size as the microscope slide (25 × 75 mm), and had a thickness between 0.5 and 4 mm, or were foil coatings on the microscope glass slide (*e.g.* nickel as 22 μm), prepared by surface thermal vapor deposition as described previously[180].

[**D**] woods and paper: common spruce wood, pine tree, maple, common beech, oak, cherry birch, lime tree, cherry tree, fir tree, apple tree, pear tree, apricot tree, peach tree, plum tree, European ash, plane tree, alder tree, larch tree, bamboo, mahogany wood. Papers: classical office paper (80 g/m^2), filter paper, and bibulous paper. All woods were rectangular pieces (25 × 75 mm) cca 5 mm thick. Papers were also cut to rectangle pieces of 25 × 75 mm and firmly mounted on the microscope glass slide using Gorilla ST352 acrylic double-sided foam metal tape (Tempus Ltd.).

[**E**] plant leaves: maple leaf, blowball (dandelion) leaf, linden leaf, acacia leaf, poplar leaf, raspberry cane, and plantain (ribwort) leaf - they were collected in the neighborhood of our research institute. All of the leaves were freshly collected as green leaves early in the summer (June) and

used immediately. The bottom side of the leaves was firmly mounted on the microscope glass slide using Gorilla ST352 acrylic double-sided foam metal tape (Tempus Ltd.), as above. Wandering larvae were distributed on the upper side (palisade mesophyll) of the leaves.

[**F**] stones (rocks) and minerals: granite, quartz, calcium aragonite, marble, gypsum, chalcedony, serpentinite, opal, clastic conglomerate, agate, ammonium alum, shale, and mica muscovite (also known as common mica or aluminum-potash mica. These materials were generously provided from the stock collection of the Geological Institute of Dionýz Štúr in Bratislava (Marián Golej), Department of Mineralogy, Petrology and Economic Geology of Comenius University in Bratislava (Peter Sečkár), and from Geopark Little Carpathians, Častá, Slovakia (Jirko Vitáloš).

[**G**] Graphene (carbon allotrope) coated on the clean microscope glass slide (25 × 75 mm) was a gift of a collaborative workshop between the Institute of Electrical Engineering of the Slovak Academy of Sciences and Danubia NanoTech Ltd. Bratislava (Martin Hulman).

[**H**] Chitins: puparial case of *Drosophila melanogaster*, elytron of recently deceased adult *Tenebrio molitor*, and pupal exuvial cuticles of *Galleria mellonella* were used as a homologous and heterologous sources of chitins, respectively.

Atomic Force Microscopy

Sgs-glue samples for atomic force microscope (AFM) measurements were prepared on cleaned standard 25 × 75 × 1 mm microscope glass slides (Langenbrinck Glasswerke GmbH., Emmendingen, BRD). An atomic force microscope (NTEGRA Prima NT-MDT) was used for all the measurements. In the semicontact mode, probeCONTV-A was used. Though the choice between the use of the semicontact and contact measuring procedures was dictated by the size and curvature of the studied surface of the sample, these provided essentially identical results. In each AFM experiment, several initial scans were made to check the reproducibility of images and the absence of possible surface irregularities or damages. The AFM images presented in this paper were performed in contact mode by a CONTV-A probe with a spring constant of 0.2 N/m. Measurements were controlled, evaluated, and imaged using NT-MDT Nova 1.1.0 and Gwyddion Image Analysis software, respectively. A minimum of five to ten independent measurements were performed for each sample.

Nanoindentation

The Sgs-glue samples were prepared on cleaned standard 25 × 75 × 1 mm microscope glass slides (Langenbrinck Glasswerke GmbH., Emmendingen, BRD) as described above for AFM. The nanoindentation analysis was made with a Hysitron TI 750D Ubi Triboindenter (Hysitron, Minneapolis, MN, USA). A Berkovich geometry diamond tip with a tip radius of ~100 nm was used. For all indents a load procedure with 5-sec loading, 2-sec hold maximal force 5000 μN, and 5-sec unloadings was applied. Three lines of indents with mutual spacing of 20 μm were made. The number of indents was calculated to analyze the full diameter of the string sample. After fitting the unloading parts of the indentation curves, hardness (H) and reduced Young's modulus (E_r) were

calculated, and expressed in gigaPascals [GPa]. The data were processed, analyzed, and visualized using the SPM multiplatform modular program Gwyddion[181].

Crystal determination

Crystals on the surface of Sgs-glue that were initially observed by AFM and SEM were further analyzed by using energy dispersive spectroscopy (EDS) and electron backscattered diffraction (EBD) equipment (Oxford Instruments) connected to a Jeol JSM 7600F SEM. The elemental and chemical analysis was then confirmed using time-of-flight secondary ions mass spectrometry (TOF-SIMS). The measurements of crystal surfaces were performed with a TOF-SIMS IV (ION TOF GmbH, Münster, Germany) equipped with a bismuth ion gun. Pulsed 25 keV Bi^+ and Bi^{3+} were used as primary ions with an ion current of 1.0 pA and 0.2 pA, respectively. The SIMS spectra were measured by scanning over a 100 × 100 μm^2 analysis area with a total primary ion dose density below the static limit of 1013 ions/cm^2.

pH measurements of the Sgs-glue and of adhesive materials

The calculated volume of the Sgs-glue released during exocytosis, followed by its continuous dilution in about 2-3 hr phase prior to expectoration, is about 20 to 25 nl per lobe, thus or the successful secretion and expectoration from a single larva, it is not expected to surpass 50 nl. To measure the pH in such an ultra-small volume presents a challenge. Standard pH electrodes widely used in research laboratories use 20 ml to liters of solute to measure pH, while the most up-to-date microelectrodes require 400 µl, which is still an 8000-fold higher volume than that produced by a larva. In addition, the expectorated Sgs-glue hardens and solidifies within a few seconds, so it cannot be collected, transferred and further manipulated as a liquid. As a first step towards this measuring its pH, we provisionally measured pH using litmus-based pH paper strips with a wide range of 1 to 14, purchased from several vendors (Machery-Nagel, Roth, Sigma, and Merck). We collected, carefully cleaned, and dried wandering third-instar larvae, and let them wander and then pupariate in 5 cm Anumbra glass Petri dishes whose bottom and sides were fully covered with a pH paper. The process of pupariation was continuously observed by one of us (DBL) under a stereomicroscope. When the larva became motionless and then expectorated its glue, it led to a small dot of rapidly changed color on the litmus paper. This was immediately compared to the color pH range provided by the vendor, and a color image was taken using a Leica MZ16F-A/X stereomicroscope equipped with an 8 MPix color camera, DFC480. The alkaline nature of the pH was evident immediately upon Sgs-glue release. This was confirmed by comparing the series of independently taken images to RGB-coded numbers for color swatches using Adobe Photoshop that were calibrated against similarly taken images of pH-color ranges provided by the litmus-paper vendor. To obtain the highest possible color accuracy, these “computer operations” were performed using a 27-inch Eizo ColorEdge CG2730 Quad-HD LCD self-calibrating monitor, and a computer having a minimum 11 GB (GDDR6) Turing GeForce EVGA RTX2080Ti or equivalent graphics card. Observations using pH-litmus indicator paper were made on groups of 20 wild-type larvae (*Oregon R*) from each of four different manufacturers, and all observations were made in triplicate. The presented value is the arithmetic mean with its standard deviation or error.

A second independent approach to estimate the pH of the Sgs-glue secretion utilized a panel of analytical pH indicators (thymol blue, *m*-cresol purple, cresol red, bromocresol purple, bromothymol blue, phenol red and congo red, purchased from Roth GmbH, Merck, and Sigma-Aldrich). Five or ten microliters of the proprietary, freshly made indicator solution was dropped on a <1 hr-old released Sgs-glue spill, and the color change was monitored and registered during the first minute. This short period of pH change monitoring was adopted following the protocol of Overend *et al.*[182] who worked with these indicators in the larval alimentary tract of *Drosophila* and observed that a color change was stable for only a few minutes.

We also developed a third method effort to measure the pH of Sgs-glue using a microelectrode, reasoning that we could estimate the pH of the glue by assessing water-extractable solutes from multiple solidified glue spills. In this approach, we applied a 5 or 10 μl drop of 18 MΩ.cm^{-1} Milli-Q (Millipore) ultrapure water over a single spill of glue that had solidified on a microscope glass slide, and let it rest for 5 min. This drop was serially transferred 10 times to additional spills. The entire process was repeated on independent glass slides a minimum of 5-times, each with a fresh 10 μl drop of Milli-Q ultrapure water, to collect a pooled 50 μl sample. Each pooled sample was diluted to a 100 μl with the ultrapure water so that we could reliably measure its pH using Thermo-Fisher Orion 9810BN microelectrode connected to a Hanna HI-223 pH-meter. These measurements confirmed pH of the glue is alkaline, as the extracted samples had pH values in the range of 8.7 to 9.1.

Although measuring the pH of solid materials is neither trivial nor widely used, there are several laboratory protocols that are based on first grinding the solid material, then mixing it with a solvent[97-99]. Using this approach solid material needs to be finely ground and mixed with a solvent to increase the solubility of extractable solutes, so that the pH is measured in the resulting slurry. In a fourth approach, we used only the purest quality water (18 MΩ Milli-Q deionized H_2O (Millipore)) as above to measuring the pH in ground-glue slurries. The pH was measured in an aliquot either by using litmus-based pH paper strips or a microelectrode as described above. We also evaluated a simpler protocol in which we applied about 300 μl of 18 MΩ Milli-Q deionized H_2O directly onto the surface of the tested material and let it extract solutes overnight in a moisture chamber, to minimize evaporation. Subsequently, its pH was measured using litmus-based pH paper strips or microelectrode, as above. The pH values obtained by these different approaches produced very similar and, in many cases, identical results.

Contact angle measurements

Contact angle (CA) measurements were performed using a OCA 25 apparatus (DataPhysics Instruments GmbH., Germany), an all-purpose contact angle measuring and contour analysis system. A dosing needle with an outer diameter of 0.31 mm and an inner diameter of 0.16 mm was used. Static CAs were obtained by dispensing of 2 μl of the test liquid on solid surface. The advancing and receding contact angles were measured by increasing and reducing the drop volume at a constant flow rate using a motorized syringe device. The Laplace-Young method was used to fit the static CA data and an ellipsoid method was used to fit the CA hysteresis data. The humidity in the laboratory was maintained within 20-30 %. All experiments were performed at temperatures between 23 and 25°C. The CA data used to calculate the surface energy were obtained using diiodomethane CH_2I_2 (purity 99%) and ethylene glycol (purity >99%); both purchased from Sigma-Aldrich Co. (USA). Water (18.2 MΩ.cm) was obtained from an AquaPro apparatus for ultrapure water production.

Surface energy calculation

For the surface energy calculation, Lifshitz-van Der Waals theory (LWAB) was used. In LWAB theory, the interfacial interaction is divided into two components: Lifshitz-van der Waals (γ^{LW}) and an acid-base component (γ^{AB}) due to electron donor/acceptor interactions according to Lewis acid-base theory.

$$\gamma = \gamma^{\mathrm{LW}} + \gamma^{\mathrm{AB}} \qquad (2)$$

Furthermore, the acid-base component γ^{AB} can be expressed as a geometric mean of the acid-base electron donor γ^{-} and electron acceptor γ^{-+} components according to:

$$\gamma^{\mathrm{AB}} = 2\sqrt{(\gamma^{+}\gamma^{-})} \qquad (3)$$

The liquid-solid interfacial energy is expressed as:

$$\gamma_{\mathrm{LS}} = \gamma_{\mathrm{L}} + \gamma_{\mathrm{S}} - 2\left(\sqrt{\gamma_{\mathrm{S}}^{\mathrm{LW}}\gamma_{\mathrm{L}}^{\mathrm{LW}}} + \sqrt{\gamma_{\mathrm{S}}^{-}\gamma_{\mathrm{L}}^{+}} + \sqrt{\gamma_{\mathrm{S}}^{+}\gamma_{\mathrm{L}}^{-}}\right) \qquad (4)$$

where γ_L and x γ_S are the liquid and solid surface free energy, respectively.

Combining the Young equation yields the following:

$$\left(\gamma_{\mathrm{L}}^{\mathrm{LW}} + 2\sqrt{\gamma_{\mathrm{S}}^{-}\gamma_{\mathrm{L}}^{+}}\right)(1 + cos\theta) \; = 2\left(\sqrt{\gamma_{\mathrm{S}}^{\mathrm{LW}}\gamma_{\mathrm{L}}^{\mathrm{LW}}} + \sqrt{\gamma_{\mathrm{S}}^{-}\gamma_{\mathrm{L}}^{+}} + \sqrt{\gamma_{\mathrm{S}}^{+}\gamma_{\mathrm{L}}^{-}}\right) \qquad (5)$$

The γ_S^{LW} x γ_S^{-} and γ_S^{+} components were calculated by simultaneously solving the above equations, using at least three different test liquids with different ratios of these components.

SUPPLEMENTAL FIGURE LEGENDS

Supplemental Figure S1. Distribution of areas of Sgs-glue fingerprints. Histograms show the distribution of area sizes occupied by (**a**) the entire Sgs-plaque and (**b**) the Sgs-fingerprint itself. Measurements were performed in 62 and 83 independent samples, respectively, using Leica LAS 3.6.1 software as described in the Materials and Methods, and shown in Figure 2.

Supplemental Figure S2. Distribution of crystal sizes of KCl on Sgs-glue plaques. The histogram shows the distribution of 106 randomly selected crystals from independent SEM images whose size was measured by using the FEI xTEM software as described in the Materials and Methods.

Supplemental Figure S3. Histograms describing the distribution of sizes of individual parts of bidentia: (**a**) main trunk; (**b**) distance between two bidentia - measured between two main trunks of the 3rd and 4th abdominal segments; (**c**) length of the anterior lateral (parameter C in Figure 6p) and (**d**) the distance between two lateral trunks on bidentium of the 4th abdominal segment. Measurements were performed in 53, 56, 58 and 53 independent SEM images, respectively, by using the FEI xTEM software as described under the Materials and Methods, and as indicated in Figure 6o.

Supplemental Figure S4. Water contact angles on evaluated substrates: (**a**) PTFE, (**b**) PLA, (**c**) polypropylene, (**d**) viton, (**e**) bakelite, (**f**) polystyrene, (**g**) kapton, (**h**) mica muscovite, (**i**) B-doped silicon, and (**j**) undoped silicon. Images of droplets were taken using OCA 25 apparatus (DataPhysics Instruments GmbH.). Insets in upper right corner show static contact angle and contact angle hysteresis values, obtained as described below in Materials and Methods.

Supplemental Figure S5. Surface free energy of tested substrates correlated with Sgs glue adhesion.

Supplemental Figure S6 On the „mechanics“ of adhesion measurements described under Materials and Methods. (**a**) Vertical (Top) view of the prepupa bearing cyanoacrylic super glue-fixed 2 mm O-ring circle (Bossard BN 726 140HV) on the dorsal side of the abdomen. (**b**) Partial side view of the prepupa with super-glue cemented O-ring circle on the dorsal side of the abdomen to document the opening throughout which the brass or copper anchor hinge (the little hook in the figure) had been passed. (**c**) Diagram of the tensometric transducer apparatus device for adhesion force measurements with a technical description of individual parts. (**A**) Front and (**B**) side view sketches. For more details see Materials and Methods. (**d**) Schematic representation of the main components of tensometric transducer apparatus device: measuring device composed of stepper motor with tensometric weight and load cell coupled via an H-bridge and HX711 processing unit to an Arduino Nano module connected to a PC via a USB port. (**e**) Photographic image of the tensometric transducer apparatus device we used in our experiments. The basic scaffold frame was constructed from hard aluminum profile blocks (grey color), whereas plastic parts of blue ABS were produced using a 3D printer. The entire device was tightly screwed to an extruded wood chipboard base (bottom).

Supplemental Figure S7 Quick guide for using ForceSketch software. (**a**) The first step after starting the program window was to open a communication connection to the computer by clicking on [Open Port] - red arrow 2. When necessary (depending on the hardware configuration), the port number was changed (*e.g.* from 3 to 7) before opening the port (red arrow 1). This depended on which USB socket in the PC is connected to the tensometric transducer apparatus. (**b**) The calibration sequence was then started by clicking on the [Calibration] button (red arrow 3) untill it stopped automatically (red arrow 4; see (**d**)), which may take ~30 sec. (**c**) Note that the metallic weight should have already been placed on the top of the tensometric balance pan before starting the calibration. In our experiments, we usually used a brass cylinder (20 × 26 mm) weighing 54.64 g. (**d**) The tested substrate (material) along with the animal was placed into the correct position on the apparatus (on the balance pan) and connected to the anchor hinge via an O-ring circle on the dorsal side of the puparium. The optimal/ideal location of this wire assembly was adjusted using the yellow and green Up and Down (full or half step) buttons (smaller black arrows). (**e**) Then, the measurement was initiated by clicking the [Start] button (red arrow 5). The steps encountered by the stepper motor usually varied from 120 to 150. (**f**) After the tension experiment was completed and the puparium was detached, the software produced the weight difference (WD) value in grams (red arrow 6), a force-time curve that recorded the entire course of the detachment experiment, and an ASCI data file that was automatically saved on the computer‘s hard disk.

Supplemental Figure S8 Spreading individual larvae on the bibulous paper, and also on PTFE can prevent their pupariation, and subsequent pupation if they do not release their Sgs-glue. However, if they find and touch each other, they will expectorate glue and stick to each other. This can result in the formation of a group of (**a**) 6 puparia, (**b**) 14 puparia, or even (**c**) 32 puparia on the PTFE surface. (**d**) to (**f**) Visible detection of alkaline pH of the Sgs-glue released on the litmus paper. To obtain SGs Sgs-glue on the litmus paper, several larvae were spread over pieces of litmus paper (in a Petri dish) and allowed to find each other, in the same way as they do on PTFE. Single larvae unable to find a favorable surface did not expectorate glue and remained larvae without pupariation, similar to how they behave on bibulous paper. Indeed, pH-litmus and bibulous papers have very similar surface structure. When two larvae were placed on litmus paper, they would only expectorate their salivary gland secretion if they contacted each other. Immediately after expectoration, the paper became wet in a dot-like region, resulting in a color change. This identified an alkaline reaction (blue spots indicated by red arrows). Even transitory contact of a “wandering” larva with another larva or prepupa was sufficient to stimulate glue expectoration. The majority of Sgs-glue lay between the two pupariating larvae, so that our observations are based on the few sets of larvae who were also in close contact with the pH-litmus paper that resulted in color change. Less often, we observed the secretion accumulated from multiple larvae (middle, up-oriented red arrow).

The different coloration seen in the puparia shown in the upper and lower rows of images results from different exposures used by the digital camera to adjust for variation in background colors of the pH-litmus papers (extreme bright white of PTFE *vs*. darker pH-litmus papers). We preferred to not manipulate image color (saturation or hue), and the images show color as determined by the exposure of the Leica digital camera.

SUPPLEMENTAL FIGURES

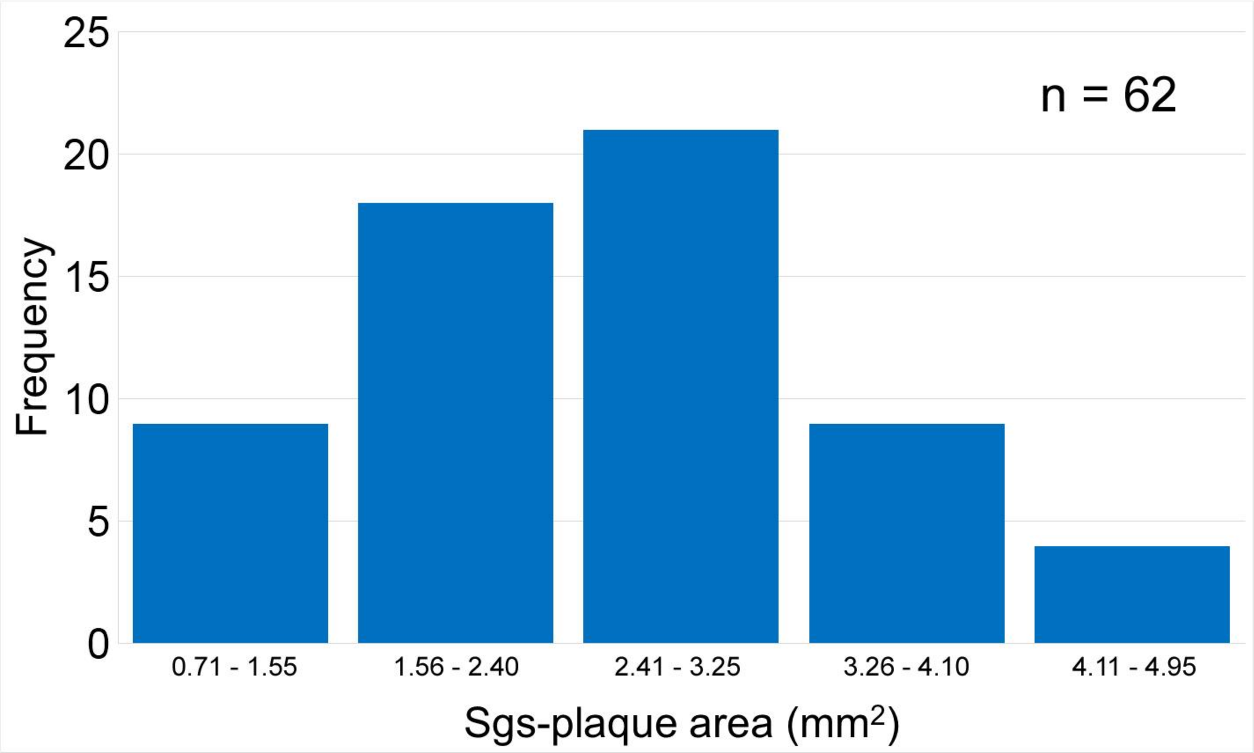

A

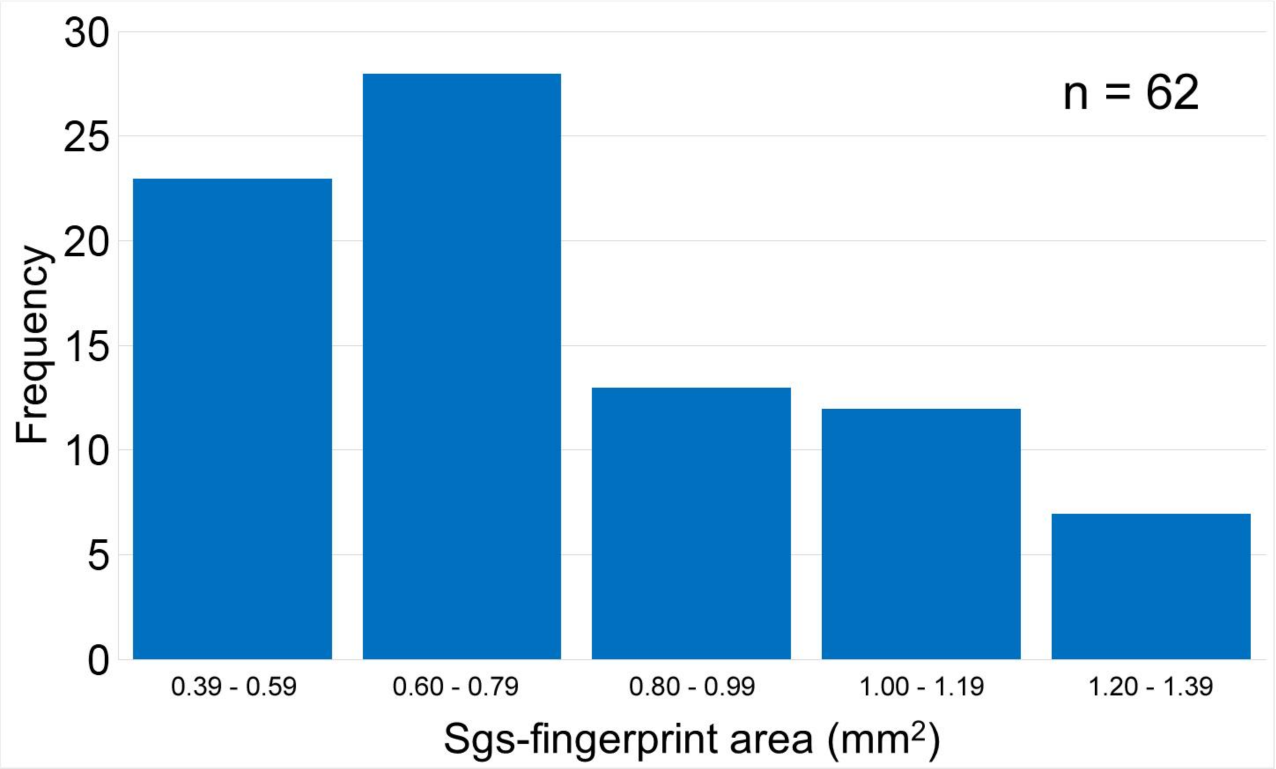

B

Supplemental Figure 1

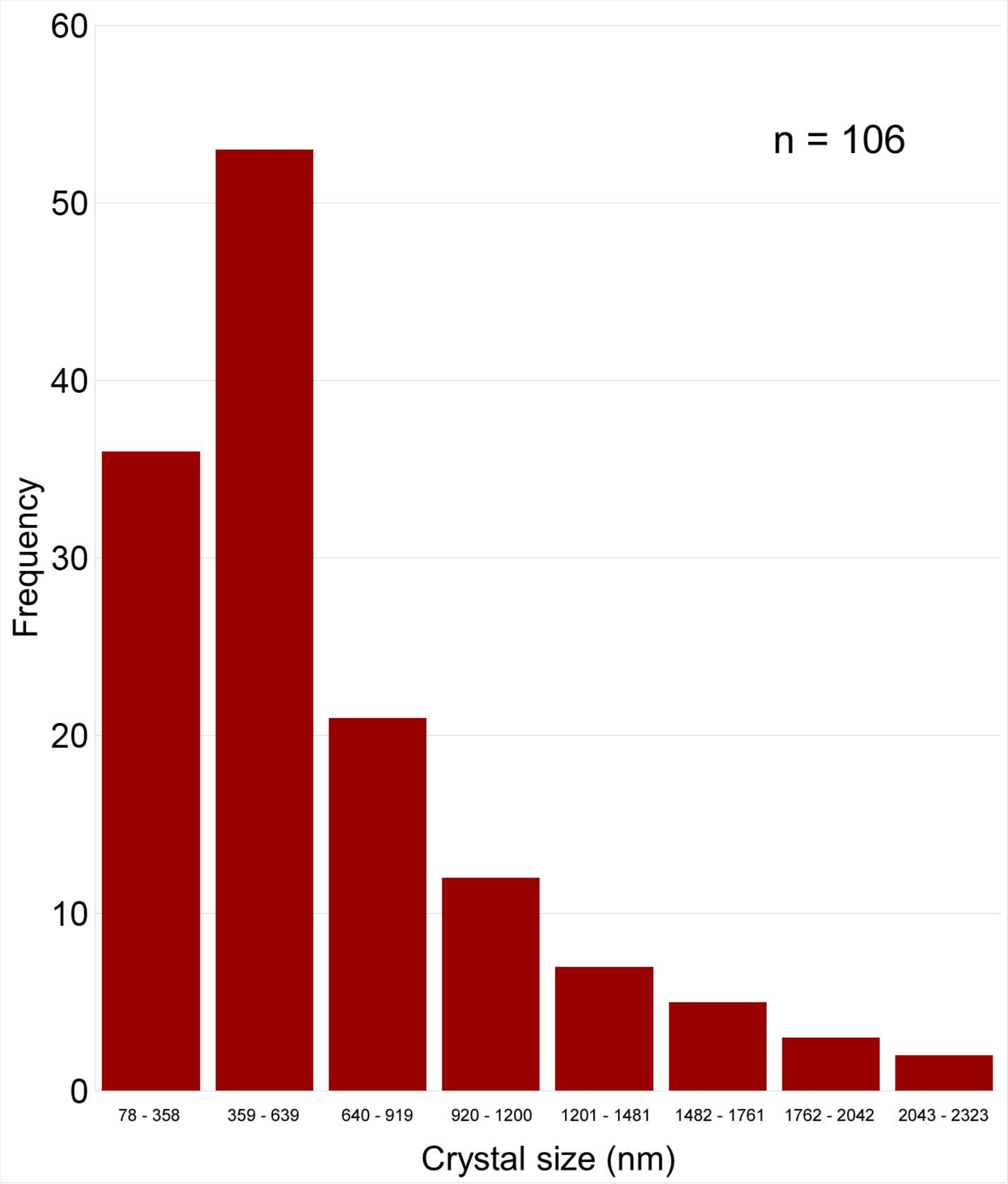

Supplemental Figure 2

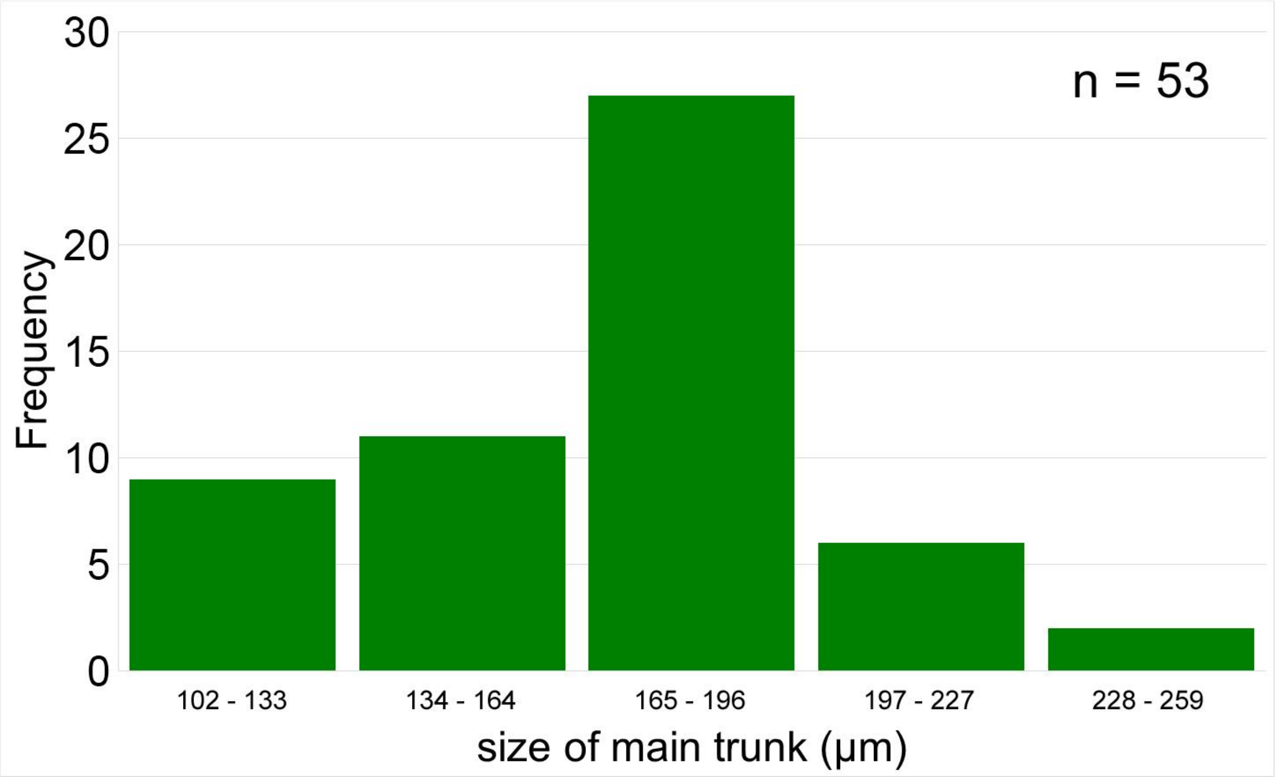

n = 53
Frequency
30
25
20
15
10
5
0
102 - 133
134 - 164
165 - 196
197 - 227
228 - 259
size of main trunk (µm)

A

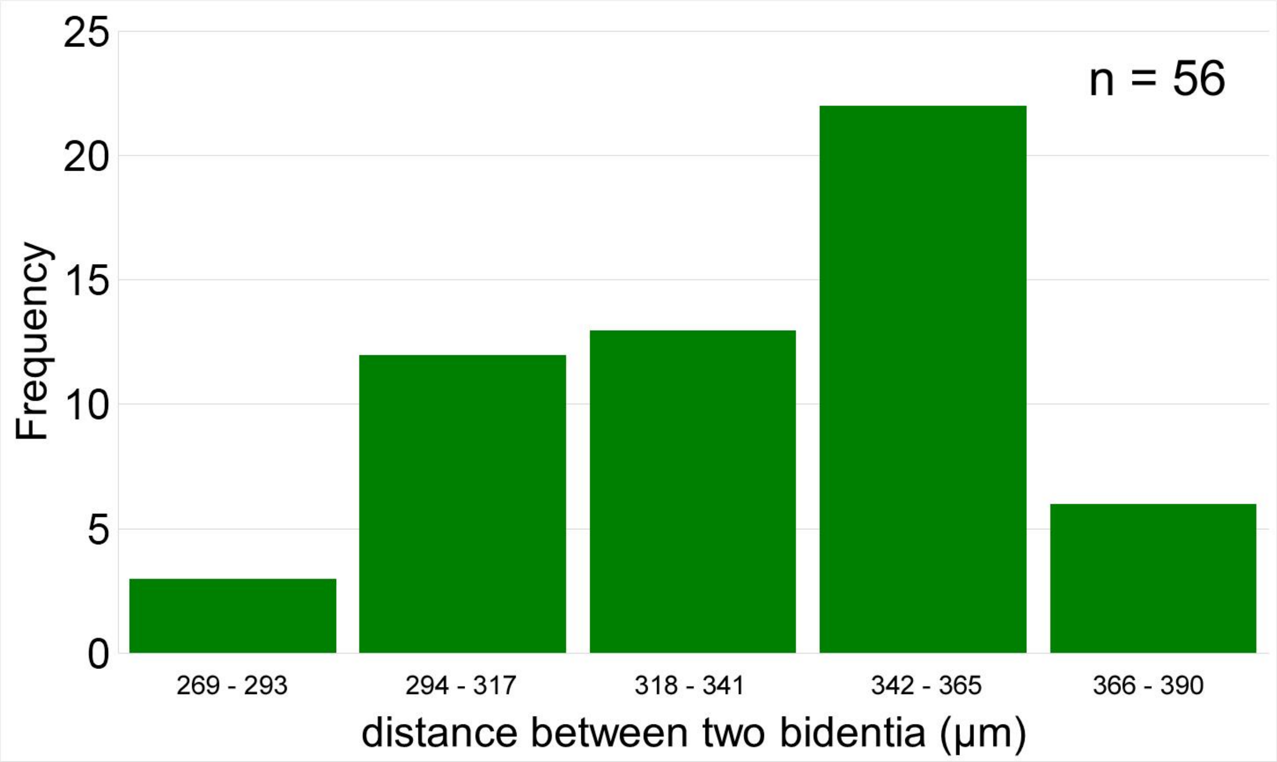

n = 56
Frequency
25
20
15
10
5
0
269 - 293
294 - 317
318 - 341
342 - 365
366 - 390
distance between two bidentia (µm)

B

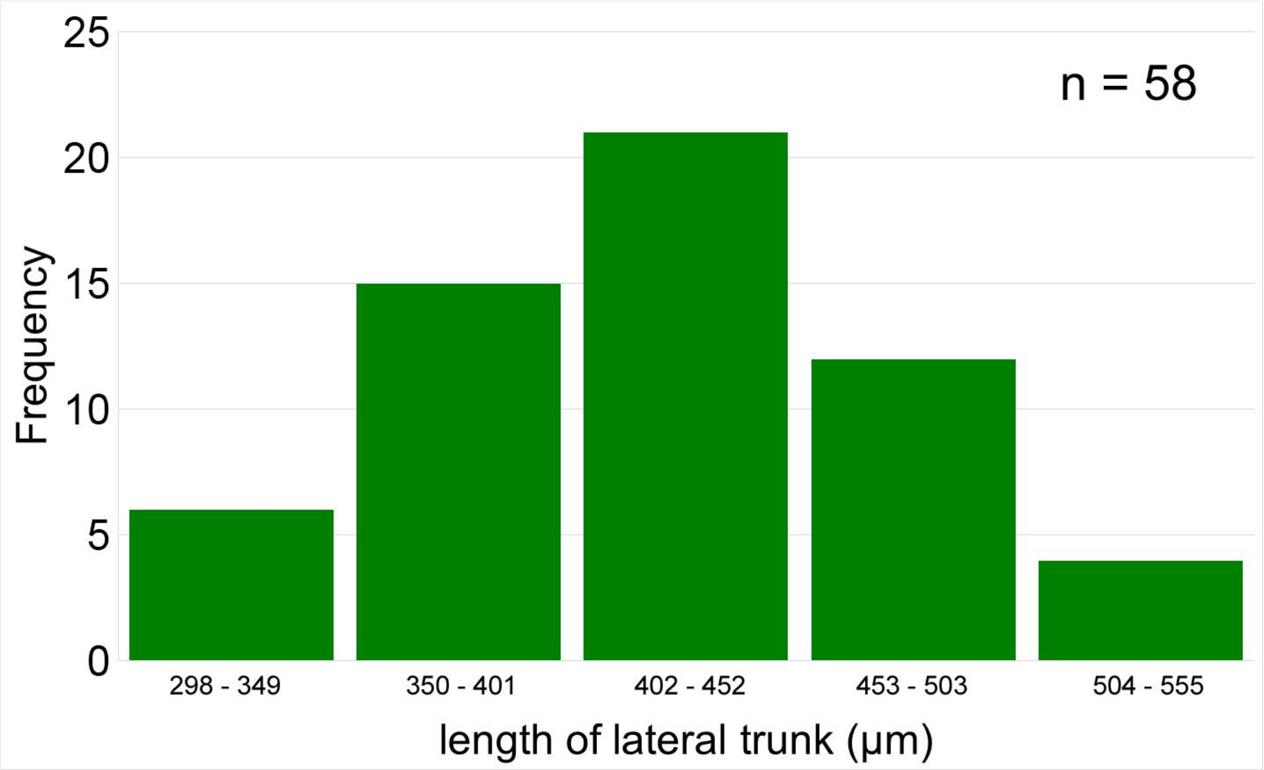

C

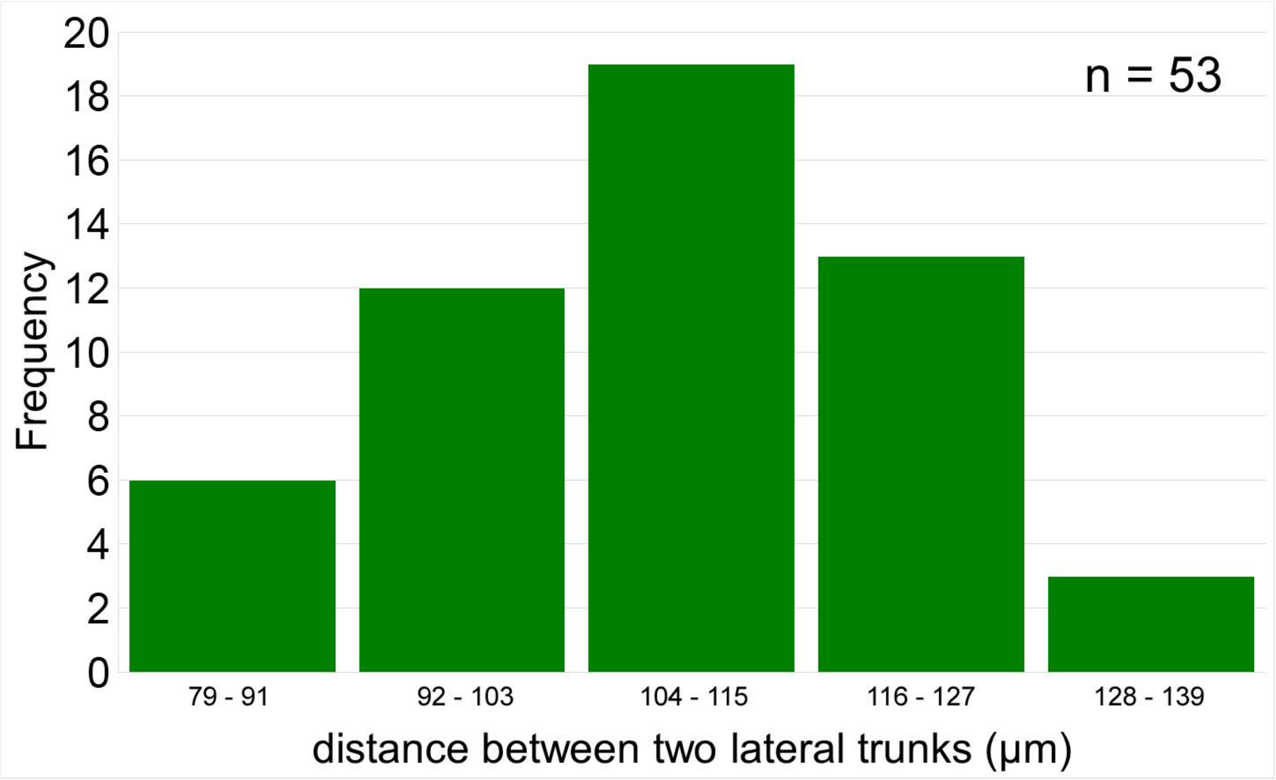

D

Supplemental Figure 3

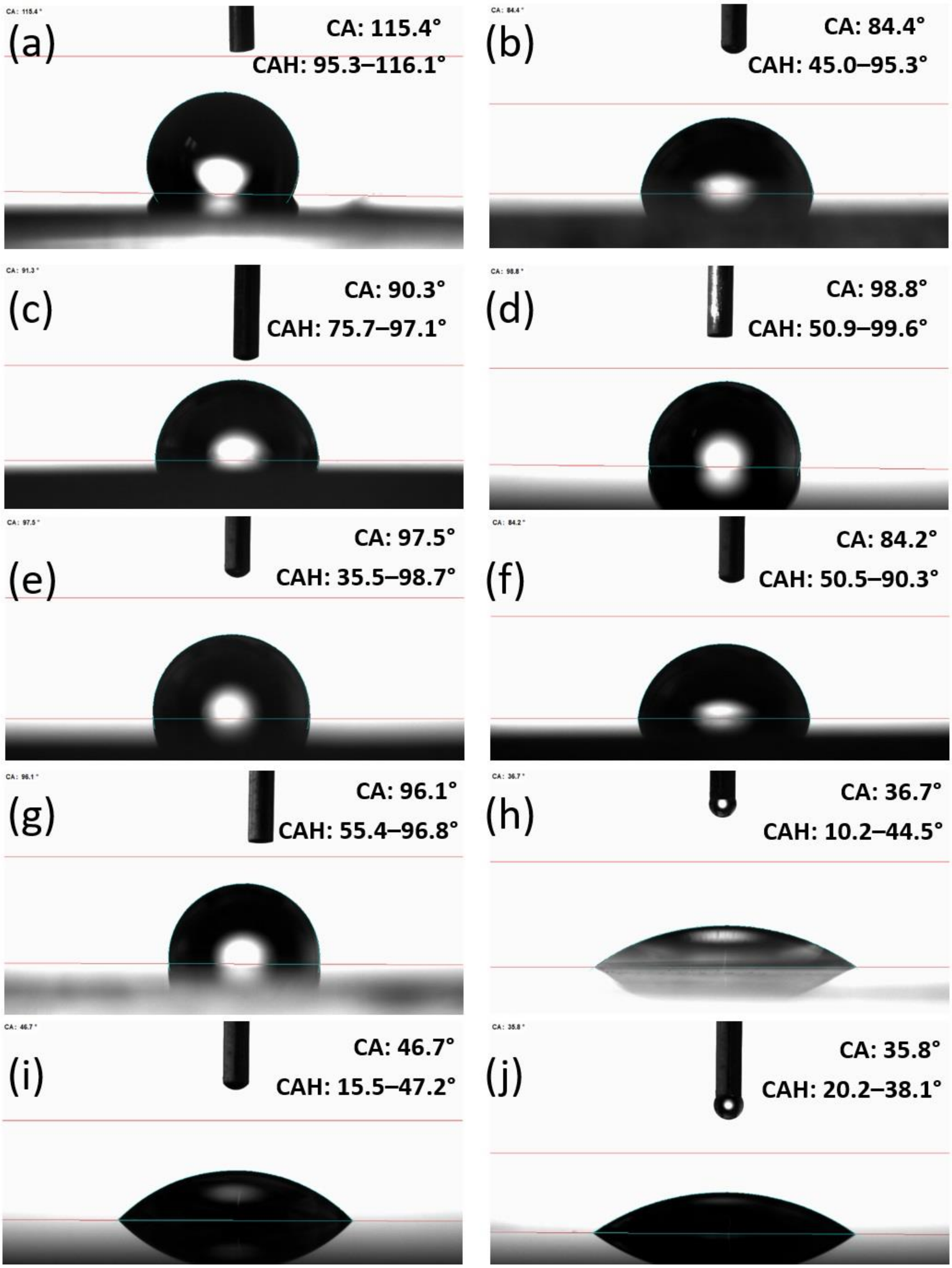

Supplemental Figure 4

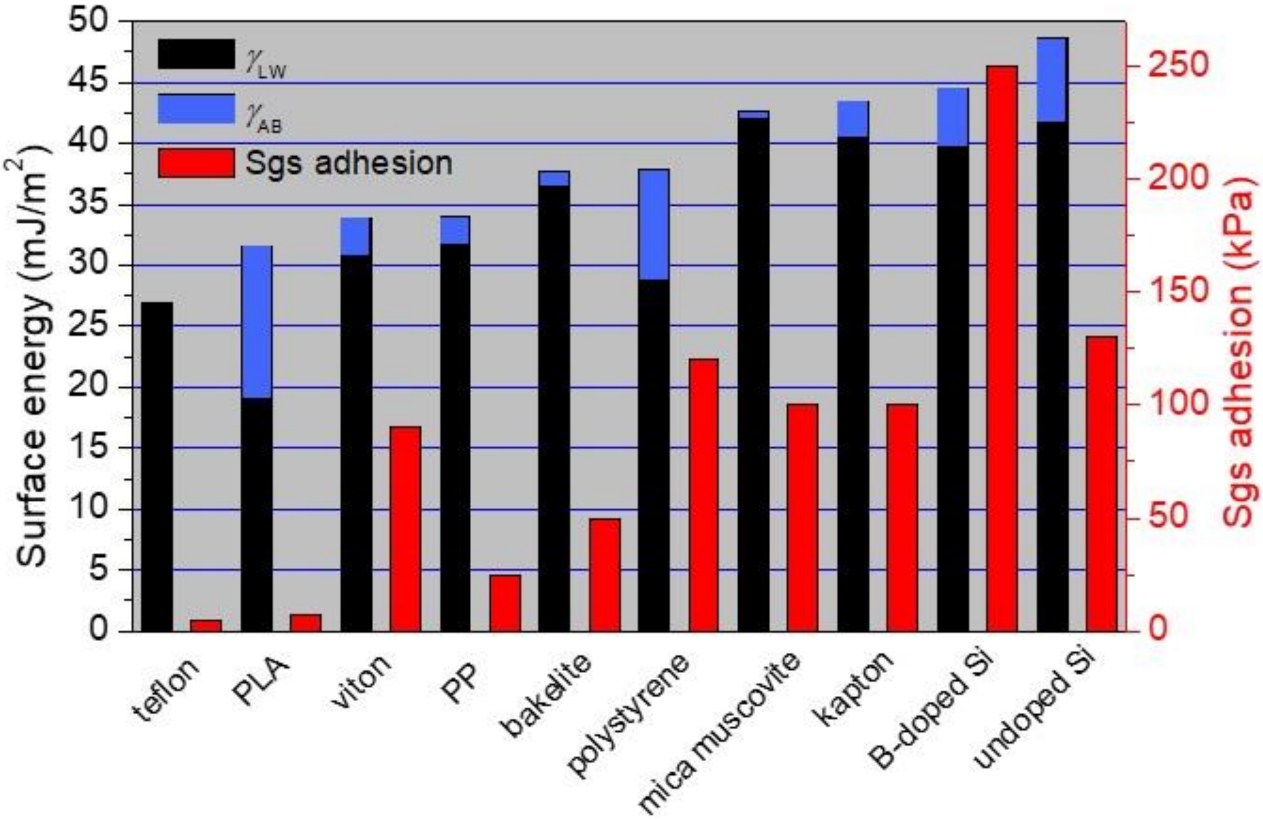

Supplemental Figure 5

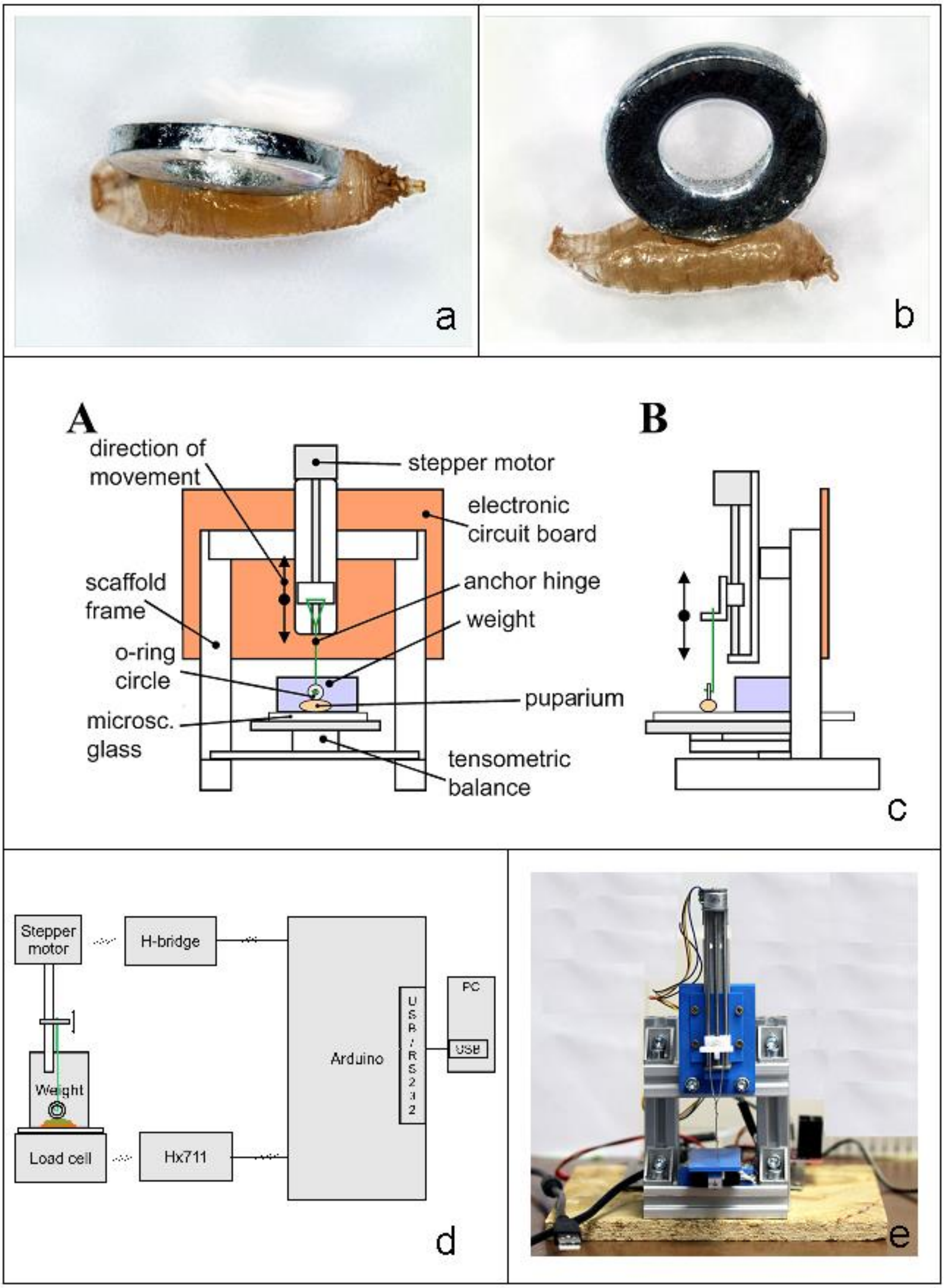

Supplemental Figure 6

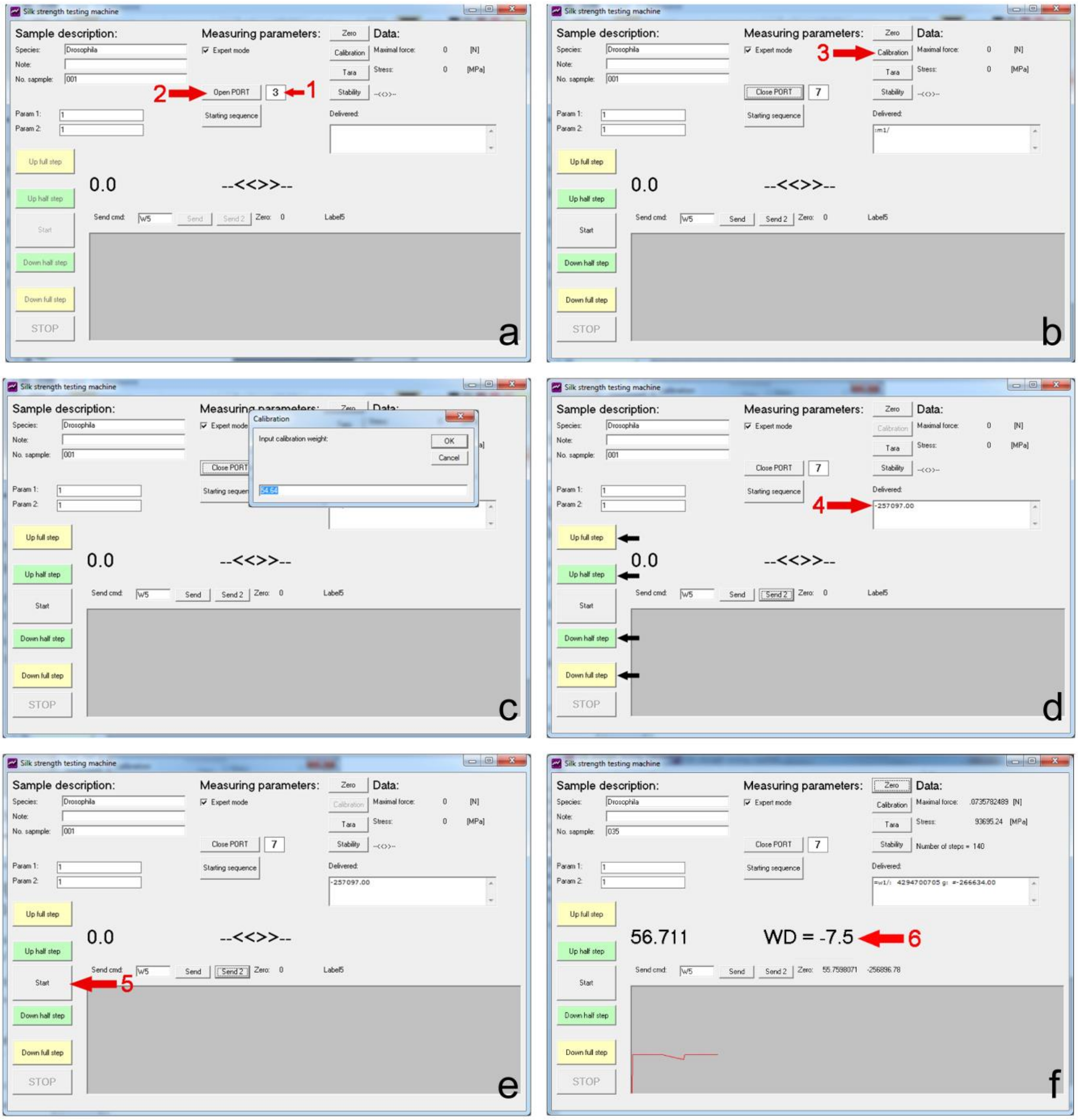

Supplemental Figure 7

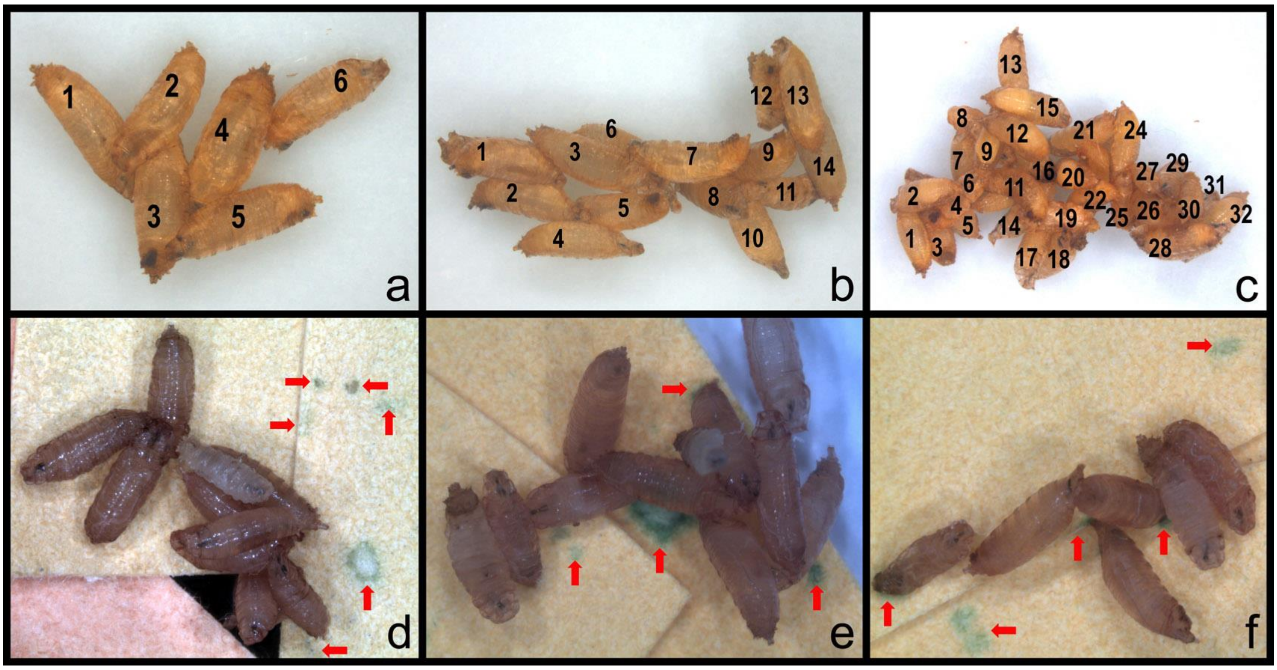

Supplemental Figure 8

SUPPLEMENTAL REFERENCES

157. Zhang, L. M. *et al*. Wettability of carbon nanotube fibers. *Carbon* **122**, 128-140 (2017).

158. Machata, P. *et al*. Wettability of MXene films. *J. Colloid Interface Sci.* **622**, 759-768 (2022).

159. Bormashenko, E., Musin, A. & Zinigrad, M. Evaporation of droplets on strongly and weakly pinning surfaces and dynamics of the triple line. *Colloids Surf. A*: *Physicochem*. *Eng*. *Asp*. **385**, 235-240 (2011).

160. Bormashenko, E. Y. Superhydrophobicity, superhydrophilicity, and the rose petal effect In *Wetting of Real Surfaces* (ed. Bormashenko, E. Y) 113-124 (De Gruyter, Berlin, Boston, 2019).

161. Huhtamäki, T., Tian, X., Korhonen, J. T. & Ras, R. Surface-wetting characterization using contact-angle measurements. *Nat*. *Protoc*. **13**, 1521-1538 (2018).

162. Marmur, A. Solid-Surface Characterization by Wetting. *Annu*. *Rev*. *Mater*. *Res*. **39**, 473-489 (2009).

163. Andrieu, C. Sykes, C. & Brochard, F. Average spreading parameter on heterogeneous surfaces. *Langmuir* **10**, 2077-2080 (1994).

164. Good, R. J. & van Oss,C. J. The Modern Theory of Contact Angles and the Hydrogen Bond Components of Surface Energies In *Modern Approaches to Wettability*. *Theory and Applications* (eds. Schrader, M. E. & Loeb, G. I.) 1-27 (Springer Verlag, New York, 1992).

165. Schrader, M. E. & Loeb, G. I. *Modern Approaches to Wettability*. *Theory and Applications* (Springer Verlag, New York, 1992).

166. Zhong, G. & Li, J. Muscovite mica as a universal platform for flexible electronics. *J. Materiomics* **6**, 455-457 (2020).

167. Drelich, J. W. *et al*. Contact angles: history of over 200 years of open questions. *Surf*. *Innov*. **8**, 3-27 (2020).

168. Ashburner, M. & Thompson, J. N. 1978 The laboratory culture of *Drosophila* In *The Genetics and Biology of Drosophila*, vol **2a** (eds. Ashburner, M. & T R F Wright, T. R. F.) 1-109 (Academic Press, London, 1978).

169. Ransom, R. 1982 *A Handbook of Drosophila development* (Elsevier Biomedical Press, Amsterdam and New York, 1982).

170. Thummel, C. S. & Pirrotta, V. New pCaSpeR P element vectors. *Dros*. *Inf*. *Ser*. **71**, 150 (1992).

171. Andres, A. J. & Cherbas, P. Tissue-specific regulation by ecdysone: Distinct patterns of *Eip28/29* expression are controlled by different ecdysone response elements. *Dev*. *Genet*. **15**, 320-331 (1994).

172. Stellwaag-Kittler, F. Zur Physiologie der Kaferhautung. Untersuchungen am Mehlkäfer *Tenebrio molitor* L. *Biol*. *Zbl*. **73**, 12-49 (1954).

173. Williams, G. J. A. & Caveney, S. Changing muscle patterns in a segmental epidermal field. *J. Embryol. Exp. Morph.* **57**,13-33 (1980).

174. Farkaš, R. The effects of 20-hydroxy ecdysone on haemolymph pressure pulsations in *Tenebrio molitor*. *J. Insect Physiol.* **30**, 797-802 (1984).

175. Sehnal, F. Kritisches Studium der Bionomie und Biometrik der in verschiedenen Lebensbedingungen gezüchteten Wachsmotte, *Galleria mellonella* L. (Lepidoptera). *Z. Wiss. Zool.* **174**, 53-82 (1966).

176. Beňová-Liszeková, D., Beňo, M. & Farkaš, R. A protocol for processing the delicate larval and prepupal salivary glands of *Drosophila* for scanning electron microscopy. *Microsc. Res. Tech.* **82**, 1145-1156 (2019).

177. Grodowitz, M. J., Krchma, J. & Broce, A. B. A method for preparing soft bodied larval *Diptera* for scanning electron microscopy. *J. Kansas Entomol. Soc.* **55**, 751-753 (1982).

178. Barbosa, P., Berry, D. & Kary, C. K. *Insect Histology*: *Practical Laboratory Techniques* (Wiley-Blackwell, Oxford, West Sussex, Hoboken, 2015).

179. Beňo, M., Liszeková, D. & Farkaš, R. Processing of soft pupae and uneclosed pharate adults of *Drosophila* for scanning electron microscopy. *Microsc. Res. Tech.* **70**, 1022-1027 (2007).

180. Matay, L. *et al.* New progressive method suitable for the exposure optimization of large and complex defect-free chips direct written by ZBA 21 e-beam tool In *ASDAM 2008 conference proceedings* (eds. Hascik, S. & Osvald, J.) 199-202 (Institute of Electrical and Electronics Engineers, Piscataway, NJ, 2008).

181. Nečas, D. & Klapetek, P. Gwyddion: an open-source software for SPM data analysis. *Central Eur. J. Phys.* **10**, 181-188 (2012).

182. Overend, G. *et al.* Molecular mechanism and functional significance of acid generation in the *Drosophila* midgut. *Sci. Rep.* **6**, 27242 (2016).
